# Ancient Genomes From Bronze Age Remains Reveal Deep Diversity and Recent Adaptive Episodes for Human Oral Pathobionts

**DOI:** 10.1093/molbev/msae017

**Published:** 2024-03-27

**Authors:** Iseult Jackson, Peter Woodman, Marion Dowd, Linda Fibiger, Lara M Cassidy

**Affiliations:** Smurfit Institute of Genetics, Trinity College Dublin, Dublin 2, Ireland; The SFI Centre for Research Training in Genomics Data Science, University of Galway, Galway, Ireland; Department of Archaeology, University College Cork, Cork, Ireland; Faculty of Science, Atlantic Technological University, Sligo, Ireland; School of History, Classics and Archaeology, University of Edinburgh, Edinburgh EH8 9AG, UK; Smurfit Institute of Genetics, Trinity College Dublin, Dublin 2, Ireland

**Keywords:** ancient pathogen genomics, oral microbiome, microbial evolution, *Streptococcus mutans*, *Tannerella forsythia*

## Abstract

Ancient microbial genomes can illuminate pathobiont evolution across millenia, with teeth providing a rich substrate. However, the characterization of prehistoric oral pathobiont diversity is limited. In Europe, only preagricultural genomes have been subject to phylogenetic analysis, with none compared to more recent archaeological periods. Here, we report well-preserved microbiomes from two 4,000-year-old teeth from an Irish limestone cave. These contained bacteria implicated in periodontitis, as well as *Streptococcus mutans*, the major cause of caries and rare in the ancient genomic record. Despite deriving from the same individual, these teeth produced divergent *Tannerella forsythia* genomes, indicating higher levels of strain diversity in prehistoric populations. We find evidence of microbiome dysbiosis, with a disproportionate quantity of *S. mutans* sequences relative to other oral streptococci. This high abundance allowed for metagenomic assembly, resulting in its first reported ancient genome. Phylogenetic analysis indicates major postmedieval population expansions for both species, highlighting the inordinate impact of recent dietary changes. In *T. forsythia*, this expansion is associated with the replacement of older lineages, possibly reflecting a genome-wide selective sweep. Accordingly, we see dramatic changes in *T. forsythia*'s virulence repertoire across this period. *S. mutans* shows a contrasting pattern, with deeply divergent lineages persisting in modern populations. This may be due to its highly recombining nature, allowing for maintenance of diversity through selective episodes. Nonetheless, an explosion in recent coalescences and significantly shorter branch lengths separating bacteriocin-carrying strains indicate major changes in *S. mutans* demography and function coinciding with sugar popularization during the industrial period.

## Introduction

The oral cavity is the most well-studied aspect of the ancient human microbiome, mainly due to the excellent preservation of DNA in calculus (fossilized dental plaque). However, three quarters of published ancient oral metagenomes date to within the last 2,500 years, with few full genomes available from prior to the medieval period (Fellows Yates et al. 2022). While a small number of much older preagricultural genomes have yielded important insights (Fellows Yates et al. 2021), prehistoric diversity and the impact of Holocene dietary transitions are not well characterized. Common oral taxa identified in these metagenomes include the “red complex” bacteria *Tannerella forsythia*, *Treponema denticola*, and *Porphyromonas gingivalis*, which are important in the development of periodontitis, a highly polymicrobial disease (Socransky et al. 1998). However, another species with a major impact on public health, *Streptococcus mutans*, is not preserved in calculus (Velsko et al. 2019), and has no published ancient genomes.

*Streptococcus mutans* is the primary cause of dental caries (Lemos et al. 2019), and is common in modern oral microbiomes (Achtman and Zhou 2020). Its lack of preservation in ancient microbiomes may be largely due to its acidogenic nature; acid degrades DNA and prevents plaque mineralization, which is the main substrate used for sampling (Velsko et al. 2019). Its absence may also reflect less favorable habitats for *S. mutans* across most of human history. Indeed, metagenomic surveys of ancient and modern microbiomes suggest that the species only became a dominant member of the oral microbiota after the medieval period due to major dietary changes, such as the popularization of sugar (Adler et al. 2013; Achtman and Zhou 2020). However, another study of modern genomes has placed more emphasis on the Neolithic transition as a driver of *S. mutans* proliferation (Cornejo et al. 2013). Caries are observed more frequently in the archaeological record after the adoption of cereal agriculture, but rise in incidence through time with a sharp increase in the Early Modern period (Bertilsson et al. 2022).

The relative importance of prehistoric and recent dietary transitions in the evolution of red complex bacteria is also poorly characterized. *Tannerella forsythia* is one of the better studied species (Warinner et al. 2014; Bravo-Lopez et al. 2020; Philips et al. 2020; Honap et al. 2023), with 55 genomes currently available (supplementary table S1, Supplementary Material online). Surveys of preagricultural (Fellows Yates et al. 2021) and European medieval diversity (Warinner et al. 2014; Philips et al. 2020) have shown no clear temporal trends in *T. forsythia* phylogenetic structure, although functional differences between ancient and modern genomes have been observed (Warinner et al. 2014; Philips et al. 2020). However, these data have not been coanalyzed. Here, we shed light on prehistoric oral pathobiont diversity, as well as recent changes in these species’ demography and functional repertoire, by retrieving the first ancient *S. mutans* genome and two distinct strains of *T. forsythia* from a single Early Bronze Age individual.

## Results and Discussion

### Ancient Metagenome Reconstruction

**Fig. 1.**
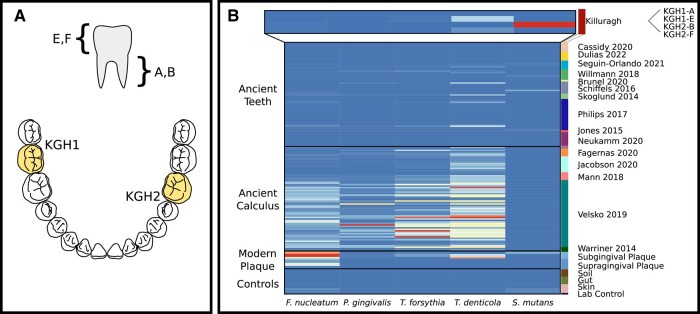
Oral microbiome retrieval from the Early Bronze Age at Killuragh Cave. a) A schematic of sampling for this study. Two teeth were sampled—KGH1 (left mandibular second molar) and KGH2 (right mandibular first molar). An aliquot was taken from each root (A and B, respectively) and each crown (E and F). b) Normalized relative abundance of 5 common oral pathobionts for ancient teeth, calculus and modern microbiome samples (Human Microbiome Project Consortium 2012; Lloyd-Price et al. 2017) and a lab control from our sequencing (see also supplementary table S3, Supplementary Material online). Relative abundance is normalized to the highest abundance sample for each taxon (*S. mutans*: 10.6%, KGH2-B; *Tr. denticola*: 4.5%, CS05; *T. forsythia*: 30.6%. CS36; *P. gingivalis*: 13.3%, CS32; *F. nucleatum:* 8.1%, SRR062298): color is scaled within species. Ancient samples are labeled and colored by publication (Skoglund et al. 2014; Warinner et al. 2014; Jones et al. 2015; Schiffels et al. 2016; Philips et al. 2017; Mann et al. 2018; Willmann et al. 2018; Velsko et al. 2019; Brunel et al. 2020; Cassidy et al. 2020; Fagernäs et al. 2020; Jacobson et al. 2020; Neukamm et al. 2020; Seguin-Orlando et al. 2021; Dulias et al. 2022).

Molars from two mandibles found in a natural limestone cave at Killuragh, County Limerick, Ireland, were sampled for aDNA analysis. These were found to belong to the same adult male individual (2,280 to 2,140 cal BC, OxA-6748; see supplementary note S2, Supplementary Material online for archaeological context). Both teeth were sampled twice: from the root (aliquots KGH1-A and KGH2-B) and the crown (KGH1-E and KGH2-F) (Fig. 1A). The metagenomic profiles of these four samples were assessed (Materials and Methods and supplementary figs. S1 and S2, Supplementary Material online) and compared with publicly available ancient sequence data from dental calculus and teeth (see dataset summary in supplementary table S2, Supplementary Material online). We found good oral microbiome preservation in both crowns and one root. In the case of the KGH1-E crown, SourceTracker2 (Knights et al. 2011) identified almost 75% of nonhost reads as deriving from the oral microenvironment: a value higher than any other tooth analyzed and comparable to that of dental calculus (supplementary fig. S1, Supplementary Material online). Two of the three members of the red complex were identified in both crowns (*T. forsythia* and *T. denticola*), as well as *Fusobacterium nucleatum* (another species involved in periodontal disease).

One root sample (KGH2-B) yielded an unprecedented quantity of *S. mutans* sequences (supplementary fig. S2, Supplementary Material online), showing a relative abundance 7.5 times higher than the next ranked samples: a medieval tooth (although realignment to a reference suggests this is a spurious result) and modern plaque (Fig. 1B, supplementary table S3, Supplementary Material online). The reasons behind this exceptional preservation are not clear, but it is likely that the cool, dry, and alkaline conditions of the limestone cave contributed favorably (Bollongino et al. 2008). Surprisingly, no evidence of dental decay was evident on the sampled tooth, although advanced caries were identified on other disarticulated teeth from the cave (Woodman et al. 2017). One possible explanation is that the individual had a systemic infection that had entered his bloodstream, which could result in the presence of *S. mutans* in the root canal. However, we note that the sample has an oral metagenomic signature with virtually no other streptococci present (supplementary figs. S1 and S2B, Supplementary Material online). This is consistent with the antagonism seen between *S. mutans* and other streptococcal species in the dental biofilm (Kreth et al. 2008). High relative abundances of S. *mutans* and a lack of other streptococcal species are an established signature of dysbiosis in modern oral microbiomes (Banas and Drake 2018; Zheng et al. 2021). Therefore, it is most likely that this genome comes from a dysbiotic biofilm prior to caries development. We note the species was virtually absent from all other ancient samples screened, with the exception of very low levels in a deeply sequenced Neolithic tooth (1H13) and an 18th century tooth with high relative abundance but shallow sequencing depth (LM_309_T) (Willmann et al. 2018; Seguin-Orlando et al. 2021).

Due to the high relative abundances of *S. mutans* and *Tr. denticola*, metagenomic assembly could be carried out, resulting in high-quality bins (see Materials and Methods and supplementary note S3, Supplementary Material online). In addition, alignment to the *T. forsythia* reference resulted in mean genomic coverages of 5.7× (KGH1-E) and 1× (KGH2-F) from the two crown samples (see supplementary table S10, Supplementary Material online for summary alignment statistics). A genotype concordance of approximately 93% between these genomes revealed the individual harbored different strains of *T. forsythia* in each tooth (see supplementary note S4, Supplementary Material online), which were analyzed separately. Alignments were also carried out for *S. mutans* (30.5×) and *T. denticola* (KGH1-E: 25.9×, KGH2-F: 1.95×). The *T. denticola* genomes were found to have high rates of heterozygosity (median: 7.7% and 11.3% for noncompetitive alignment; 4.1% and 4.6% for competitive alignment, respectively). This is likely due to high treponemal diversity in the oral cavity (Zeng et al. 2021). Even higher heterozygosity rates were seen in downloaded ancient datasets, which prevented further downstream analysis for this species (see supplementary note S5, Supplementary Material online).

### *T. forsythia*: Postmedieval Reduction in Diversity and Virulence Acquisition

**Fig. 2.**
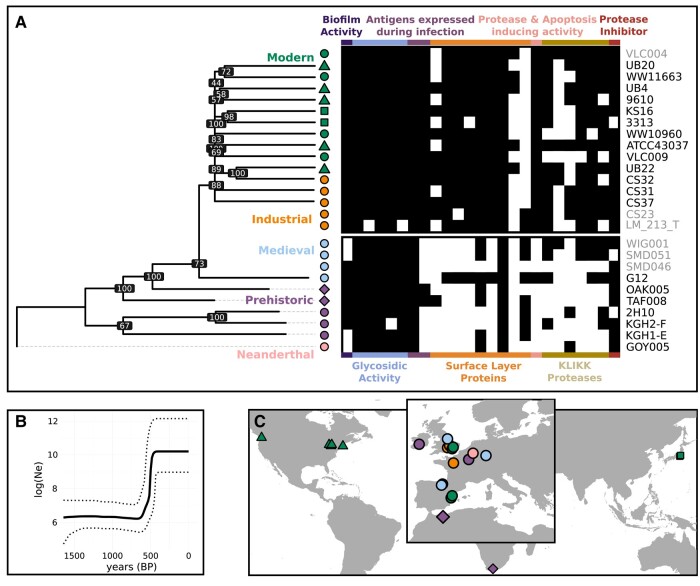
*Tannerella forsythia* phylogenetic structure and virulence profiles. a) Maximum likelihood (ML) tree of *T. forsythia* sequences, using a Neanderthal sequence as an outgroup, alongside a matrix showing presence (black) or absence (white) of virulence genes examined by Philips et al. (2020), with sample IDs and symbols aligned with their positioning on the ML tree. Colors and shapes reflect time period and continental region, respectively. Grayed-out sample IDs were excluded from the main tree due to low coverage and are positioned with respect to time period. Trees with these additional 3 medieval and 2 industrial samples are shown in supplementary figs. S13 to 15, Supplementary Material online supporting the temporal structure seen here. Virulence categories are shown along the *x* axis in different colors. b) Bayesian skyline plot from BEAST showing inferred population size over the last 1,500 years, illustrating an exponential increase starting approximately 1,400 CE. c) Sampling locations of the genomes included (Europe and North Africa inset).

We assembled a time transect of *T. forsythia* genomes, spanning key archaeological and historical transitions. This includes 10 modern genomes, the two genomes from Killuragh, as well as 13 ancient genomes reconstructed from publicly available data, of which only four have been previously subject to phylogenetic and functional analysis. With this dataset, we find that *T. forsythia* diversity can be clearly split into preindustrial and postindustrial groupings on the basis of both phylogenetic structure and the presence of key virulence factors (Fig. 2A).

First we subsetted our dataset to maximize sample age range and the number of SNPs for analysis (see Materials and Methods and supplementary note S1, Supplementary Material online). We then demonstrated, using ML trees with a dog-derived strain as the outgroup, that a Neanderthal strain of *T. forsythia* forms an outgroup to all *Homo sapiens* strains. Another ML tree was then constructed, rooting on the Neanderthal sample, which showed strong temporal structure (Fig. 2A). We observed the prehistoric strains to be basal to all samples from the medieval period onwards, and single medieval genome to outgroup industrial and modern diversity. To achieve higher resolution over the past 1,000 years, we added 5 additional lower coverage medieval and industrial genomes to our dataset, and either removed all premedieval samples (supplementary figs. S13 and S14, Supplementary Material online) or all premedieval samples below 5× coverage (supplementary fig. S15, Supplementary Material online). We found all industrial and modern samples from the USA, Japan, and Europe to form a clade within European medieval diversity in both Bayesian and ML trees (supplementary figs. S13 to S15, Supplementary Material online). This imbalanced structure has been previously seen for phylogenies of pathogens sampled from different eras (e.g. *Yersinia pestis*) (Spyrou et al. 2019; Guellil et al. 2020) and suggests repeated lineage expansions replacing older diversity. These homogenizing events appear to have intensified in recent history, as we find that the 2 Killuragh strains from the same mouth are more divergent from each other than any pair of postmedieval samples (Fig. 2A). Indeed, KGH2-F forms a clade with a Neolithic French genome (3,341 to 3,098 cal BC) to the exclusion of KGH1-E, implying a continuation of Neolithic *T. forsythia* diversity into the Bronze Age in Europe.

We also find evidence for a rapid expansion in *T. forsythia* population size beginning approximately 600 years before present (Fig. 2B; supplementary fig. S13, Supplementary Material online), inferred from Bayesian skyline analyses from 2 independent BEAST runs of genomes from the medieval period onwards (Materials and Methods). A combination of factors is likely responsible, including changes in host demography and population size. We also observe an uptake in virulence factors across this time interval (Fig. 2A), indicative of a major adaptive episode driven by changes in the oral microenvironment. We find medieval and prehistoric virulence factor profiles are significantly depleted relative to later genomes (Materials and Methods and supplementary tables S4 and S5, Supplementary Material online). This distinction is most pronounced for surface layer (S-layer) associated proteins, antigens expressed during infection, and genes associated with protease- and apoptosis-inducing activity. There are also fewer KLIKK proteases in medieval genomes (previously noted by Philips et al. 2020) and prehistoric genomes, although some medieval genomes closely resemble industrial and modern ones in their KLIKK protease repertoire. The medieval sample G12 also has an S-layer protein profile more similar to moderns, as noted by Warinner et al. (2014), suggesting that certain functional acquisitions had occurred by this period.

The rapid uptake of new S-layer proteins likely reflects changes in oral ecosystem composition over the past several 100 years (Adler et al. 2013), as these proteins mediate interactions with other oral microbes. Shimotahira et al. (2013) demonstrate that coaggregation is inhibited in S-layer deficient mutants. S-layer proteins are also key players in the arms race between host and pathogen; they are responsible for gingival cell invasion and disease initiation (Sakakibara et al. 2007) and are recognized by the adaptive immune system of periodontitis patients (Yoneda et al. 2003). Thus, these changes in the virulence repertoire would have had wide-reaching impacts on both oral biofilm ecology and the host immune response.

### *S. mutans*: Persistence of Prehistoric Diversity and Recent Population Expansions

We report here the first ancient *S. mutans* genome, analyzed in tandem with a dataset of 410 modern genomes. As *S. mutans* undergoes extensive recombination, these data were first filtered to remove inferred recombinant regions using the tool Gubbins (Croucher et al. 2015). Bayesian and ML phylogenetic trees reveal that deeply divergent (pre-6,000 BP) lineages exist in modern *S. mutans* populations, a sharp contrast to what we observe in *T. forsythia*. KGH2-B is among the earliest branching lineages (median estimated TMRCA with another taxon in the tree: 25,736 BP; 95% HPD: 5,804 to 160,951), while still falling within modern diversity. We can partition our phylogeny into 7 major clades (earliest branchings with >90% posterior support), with the root node dating to 32,049 BP (95% HPD: 6,615 to 202,100). These dates align with the expansion of *H. sapiens* across the globe; however, we cannot preclude these clades originating in the Neolithic period, given the wide confidence intervals. We also note the lack of African diversity in our dataset (supplementary table S6, Supplementary Material online).

Previous work with 56 modern genomes inferred a population expansion coinciding with the origin of cereal agriculture (Cornejo et al. 2013). The increase in sample size, as well as the inclusion of the 4,000-year-old genome reported here, indicates that multiple expansions of *S. mutans* lineages occurred after the medieval period. The number of coalescences in the tree increases from about 4,000 years ago, reaching a peak between 250 and 750 years ago. Samples coalescing in the last 250 to 1,000 years tend to group by continental region, while clades that have diversified more recently are often restricted to 1 country; of the 42 groups that diversified in the past 250 years, excluding those with samples of unknown origin, just 17 have more than 1 country represented (supplementary figs. S17 and S18, Supplementary Material online). This coincides with sugar becoming a large component of people's diets from the 18th century onwards, although it initially radiated from domestication centers over 3,000 years ago (Grivet et al. 2004). High sucrose levels allow *S. mutans* to thrive; it uses sucrose to synthesize glucans, which are important in biofilm formation and contribute to a low pH, giving this acid-tolerant species an advantage.

**Fig. 3.**
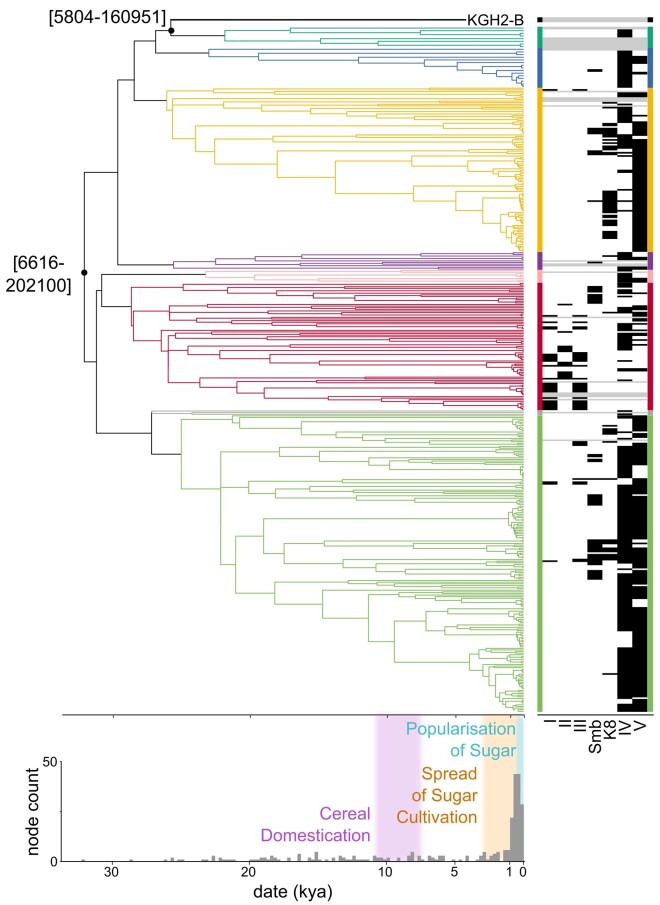
*Streptococcus mutans* phylogeny and mutacin profiles. Phylogeny inferred using MCC tree based on median values from independent BEAST runs of 500 million chains each. The deepest clades with >90% posterior support are colored (seven total). KGH2-B (black) and three modern genomes (gray) do not reliably group with any others. Eight nodes had median values lower than their direct descendent nodes, resulting in negative edge lengths, which were set to zero for visualization purposes only. A presence/absence matrix of 7 mutacins investigated in Watanabe et al. (2021) is aligned to tree tips. Black indicates mutacin positive. Samples lacking any mutacins are highlighted in gray and tend to have long branches. Underneath the phylogeny, median node heights from the BEAST tree are plotted in 250 year bins, showing a sharp increase in coalescences in the last 750 years. Eras of major dietary change are highlighted: cereal domestication in the Near East (Allaby et al. 2017); sugarcane spread from centers of domestication (Grivet et al. 2004) and the popularization of sugar in the 18th century.

A principal factor in *S. mutans* virulence are mutacins. These are bacteriocins (peptide antibiotics) specific to *S. mutans* that allow the species to outcompete other streptococci during biofilm formation by inhibiting their growth, including the noncariogenic *Streptococcus gordonii* and *Streptococcus sanguinis* (Merritt and Qi 2012; Watanabe et al. 2021). *Streptococcus mutans* is exceptional within the streptococcal genus for bacteriocin production, and it has been hypothesized that mutacins are essential for survival (Merritt and Qi 2012). Less than 7% of our dataset (26 genomes) have no detectable mutacins (gene list from Watanabe et al. 2021), including KGH2-B and many of the deeply divergent strains that group with it (Fig. 3). We find that phylogenetic placement informs mutacin profile to an extent. Specifically, mutacin-positive strains on average share common ancestors with their neighbors significantly more recently than mutacin-negative strains (supplementary note S1 and supplementary fig. S19, Supplementary Material online). This suggests that mutacin acquisition has played a role in *S. mutans* population expansions over the past millennium. Accordingly, *S. mutans* is one of the only oral taxa to show a significant increase in abundance between medieval and modern microbiomes (Adler et al. 2013).

Additional ancient *S. mutans* genomes would allow the timing of mutacin acquisition to be better characterized. Although this is the first complete *S. mutans* genome, low quantities of *S. mutans* have been previously reported, including in a French Neolithic tooth (1H13) (Seguin-Orlando et al. 2021), for which we reconstruct a 0.4× genome for the first time. This sample was aligned to the pangenome estimated using the 410 modern assemblies and KGH2-B. No reads were found covering any of the inferred mutacin genes, a result consistent with these genes being few in number or absent entirely in 1H13. This conclusion is supported by low coverage simulations (see supplementary note S7, supplementary fig. S20 and supplementary table S7, Supplementary Material online) and is in keeping with our hypothesis that extensive mutacin acquisition is a more recent phenomenon. We note that 1 mutacin gene was observed in another newly constructed genome from an 18th century sample (LM_309_T, 0.4×) (Willmann et al. 2018).

## Conclusion

Adding a temporal dimension to pathogen genomics allows us to better estimate the timing of key evolutionary changes, as well as to capture extinct diversity. In this study, we have reconstructed ancient genomes for *T. forsythia* and *S. mutans*, which demonstrate dramatic changes in oral pathobiont population dynamics and functional composition in the last 750 years. For both species, there is a distinction between postindustrial and earlier genomes in terms of virulence factors. This is clearest in *T. forsythia*, where there is a temporal transect of ancient genomes: here, preindustrial genomes have a stark difference in functional repertoire compared to industrial and modern genomes. Both the host immune response and interactions with other oral microbes would be impacted by these changes. Although there is only 1 ancient *S. mutans* genome (KGH2-B) of sufficient quality to compare with the modern dataset, analysis in tandem with phylogenetic information implies that the modern mutacin repertoire is also a relatively recent acquisition.

Concurrent population expansions were inferred in both *S. mutans* and *T. forsythia* phylogenies in the postmedieval period, but extant *S. mutans* populations harbor much deeper diversity compared to *T. forsythia*. We hypothesize that this is related to the species’ different susceptibilities to genome-wide selective sweeps, which are more likely to occur when within-population recombination is low (Bendall et al. 2016). *Streptococcus mutans* is a highly recombining species (Cornejo et al. 2013), allowing advantageous variation to be exchanged between population members and resulting in gene-specific sweeps. In *T. forsythia*, genome structure is relatively stable and small-scale mutation appears to be the major driving force of diversification (Endo et al. 2015). This could lead to repeated purges in genetic heterogeneity in the population. These purges may have intensified in the past several centuries, as evidenced by the loss of diversity in modern and industrial populations, relative to prehistoric strains from the same Early Bronze Age individual. In general terms, higher biodiversity in ecosystems makes them more resilient to perturbations from environmental stressors (e.g. dietary changes and colonization by pathogenic bacteria in the case of the oral microbiome). The reduction in *T. forsythia* diversity over time coincides with a general loss of oral biodiversity discussed in Adler et al. (2013).

Going forward, denser sampling of ancient microbial populations may provide new insights into the evolutionary mechanisms that underlie bacterial taxon formation, adaptation and maintenance of diversity, which can vary depending on species and environment. Some species will be more amenable to dense temporal sampling than others. In particular, low levels of *S. mutans* preservation in the ancient DNA record may pose a significant challenge. Targeted capture of genes of interest (e.g. mutacins) as well as the core genome may provide a solution, but risks losing pangenomic diversity. In the case of *T. forsythia,* which is more abundant in the archaeological record, ancient metagenomic assemblies in the future may allow for the identification of hitherto unknown virulence factors in earlier strains; our analysis here is limited to the genomic content of modern *T. forsythia* samples, as all the ancient genomes are reference-aligned.

Overall, our findings demonstrate the utility of ancient genomes in characterizing different modes of pathobiont evolution. Temporal resolution of virulence genes can provide further insight into the shifting selection regimes of pathobionts in the human oral microbiome. In addition, these results highlight that recent cultural transitions, such as the popularization of sugar, are most relevant to understanding the shaping of present-day oral pathobiont diversity.

## Materials and Methods

For detailed descriptions of sampling, sequencing, data processing, and analysis, see supplementary note S1, Supplementary Material online.

### Sampling and Sequencing

Both teeth were cleaned mechanically and exposed to 30 min of UV radiation on either side. For each tooth, pulp was removed prior to sampling a section of root (KGH1-A, KGH2-B) and crown (KGH1-E, KGH2-F). KGH1-A, KGH2-B, and KGH2-F were subject to the same extraction protocol as in Cassidy et al. (2020) and described in the extended methods. The protocol for KGH1-E differed slightly, in that the first 2 incubations were in an EDTA solution and only lasted 30 min. Non-USER treated Illumina libraries were created for KGH1-A, KGH2-B, and KGH2-F as in Cassidy et al. (2020) following the Meyer and Kircher (2010) protocol and screened at a low read depth on an Illumina Miseq platform (Trinseq, Trinity College Dublin; 65 bp single-end reads). All 4 extracts were treated with USER enzyme and sequencing libraries were created as above. KGH1-A, KGH2-B, and KGH2-F were sequenced on an Illumina Hiseq 2500 (Macrogen, 100 bp single-end reads). KGH1-E was screened on an Illumina Novaseq 6000 (Biosource, 150 bp paired-end reads) and sequenced to a high read depth on an Illumina Novaseq 6000 (Trinseq, Trinity College Dublin) with 50 and 100 bp paired-end reads.

### Data Processing

Residual adapters were trimmed from raw sequencing reads in FASTQ format using cutadapt v1.2.1 for single-end reads and AdapterRemoval v2.2.2 for paired-end reads (Martin 2011; Schubert et al. 2016), and paired-end reads with more than 11 bp overlapping were collapsed. Collapsed reads only were used for downstream analysis. Trimmed reads were aligned to the human reference hs37d5 using bwa aln with relaxed parameters (Li and Durbin 2009), and unaligned reads were converted back to FASTQ and used for metagenomic analysis. These files were fished for exact index matches, deduplicated with prinseq++ (Cantu et al. 2019), and merged to sample level. An identical approach was used for downloaded published data, without the index matching.

### Metagenomic Analysis

Taxonomic profiling was performed using kraken2 (Wood et al. 2019), with the NCBI RefSeq database for bacteria, archaeal and viral sequences, and these assignments were refined using Bracken, with average read length set to 65, and threshold set to 100 (Lu et al. 2017). OTU tables were generated using kraken-biom (Dabdoub 2016). SourceTracker2 was used to compare taxonomic composition of the ancient data with modern microbiome data, soil data, and lab controls (Knights et al. 2011).

Metagenomic assembly was carried out on the deeply sequenced libraries for KGH1-E and KGH2-B. Contigs were assembled from FASTQ files using MEGAHIT (Li et al. 2015). Contigs were split to a maximum length of 10 kB and reads were mapped back to the assembled contigs for binning with CONCOCT (Alneberg et al. 2014). Resulting bins were assessed with CheckM and GTDB-Tk (Parks et al. 2015; Chaumeil et al. 2019).

### *S. mutans* Pangenome

The *S. mutans* pangenome was calculated from published modern genomes (supplementary table S6, Supplementary Material online) and the KGH2-B metagenome-assembled genome (MAG) using roary v.3.12.0 (Page et al. 2015). Genomes were annotated using PROKKA 1.14.6, using the coding sequences annotated in the UA159 assembly as a training set (Ajdić et al. 2002; Seemann 2014). Each coding sequence in the pangenome was compared with the NCBI nr database using diamond blastx (Buchfink et al. 2021), and those matching the mutacin sequences in Watanabe et al. (2021) were annotated as mutacins.

### *S. mutans* Phylogenetic Analysis

Multiple sequence alignment for modern *S. mutans* and the KGH2-B MAG was carried out with SKA (Split Kmer Analysis) with the UA159 sequence as a reference (Harris 2018). Recombinant regions were filtered with Gubbins (Croucher et al. 2015), and any residual identical sequences were removed. SNP sites were extracted using snp-sites (Page et al. 2016), and were filtered for biallelic sites and maximum missingness of 5%, leaving 7,629 variant sites.

IQTree was used to construct a maximum likelihood tree, using -m MFP to select the best-fitting model for sequence evolution (GTR + F + ASC + R5) and 1,000 rapid bootstraps (Kalyaanamoorthy et al. 2017; Hoang et al. 2018; Minh et al. 2020).

Bayesian phylogenetic analysis was also carried out using BEAST v2.6.7 (Bouckaert et al. 2019). Invariant sites were specified in the BEAST XML. The site model was defined with BEAST Model Test, and a Bayesian skyline prior and relaxed lognormal clock was used (Drummond et al. 2005, 2006; Bouckaert and Drummond 2017). Two independent runs with 500 million chains and 1 million burnin steps converged and were combined using LogCombiner. The maximum clade credibility tree was calculated using TreeAnnotator from median heights, removing burnin trees from the first 10 million chains.

### Reference Genome Alignment

FASTQ files were aligned to appropriate reference genomes (supplementary table S8, Supplementary Material online) using bwa aln with relaxed parameters (-l 165,000 -n 0.01 -o 2) (Li and Durbin 2009). BAM files were filtered for duplicate reads with Picard MarkDuplicates, mapping quality > 25 and read length > 34. For USER-treated samples, the base quality of the first and last 2 bases was reduced to zero to mitigate postmortem damage, which was increased to 5 for non-USER-treated samples. Depth and breadth of coverage was calculated with qualimap v2.2.1 (Okonechnikov et al. 2015), and the shape of the edit distance distribution was summarized as in the HOPs pipeline (Hübler et al. 2019). For *T. forsythia,* modern genomes were downloaded from Genbank as FASTA files. In order to make these comparable to ancient alignments, pseudo-reads of length 100 bp and a slide of 1 base were generated and aligned to the same reference as the ancients, as in Philips et al. (2020). These alignments were filtered for minimum mapping quality 25.

### SNP Ascertainment and Calling: *T. forsythia* and *Tr. denticola*

SNP sites were ascertained using modern genomes and ancient samples with coverage > 2×. Sites were called using GATK's UnifedGenotyper in discovery mode, with minimum base quality 30 (McKenna et al. 2010). SNPs were filtered for coverage within 2 standard deviations of mean genomic coverage, and with a hard minimum of 2 reads supporting a genotype call. Sites flagged as low quality by GATK were also removed. These sites were called in all genomes >1× using GATK pileup (McKenna et al. 2010), filtering for base quality > 30, mapping quality > 25, read length > 34 and a consensus of at least 2 reads. Heterozygosity at these variant sites in the pileup was also assessed, as was identity between the two Killuragh genomes (see supplementary note S1, Supplementary Material online).

### *T. forsythia* Phylogenetic Analysis

ML trees for *T. forsythia* were constructed with IQTREE using sequences with < 40% SNP missingness; this filter was relaxed to include GOY005, TAF008 and KGH2-F, as well as the dog-derived outgroup OH2617_COT023 for initial analyses, with ModelFinder to assess models of sequence evolution and 1,000 rapid bootstraps as in *S. mutans* (Kalyaanamoorthy et al. 2017; Hoang et al. 2018; Minh et al. 2020).

All genomes with >1× mean coverage from the medieval period onwards were used to assess the inferred *T. forsythia* expansion using BEASTv2.6.7 (Bouckaert et al. 2019). The temporal signal was assessed using maximum likelihood trees and TempEst (see supplementary note S1, Supplementary Material online) (Rambaut et al. 2016). SNP data were filtered for maximum missingness of 5% and partitioned to codon positions 1, 2, 3 and noncoding regions. These data were analyzed under a Bayesian skyline model with a relaxed lognormal clock with BEASTv2.6.7 (Bouckaert et al. 2019). bModelTest was used to assess the best model for sequence evolution and invariant sites were specified in the XML. Two independent runs with 300 million chains each and a 3 million preburnin step were performed. Maximum clade credibility trees and Bayesian skyline plots were constructed using a 10% burnin.

### *T. forsythia* Virulence Factors

Bedtools genomecov was used to estimate coverage across *T. forsythia* virulence factors (coordinates from Philips et al. (2020) and Quinlan (2014). Reads were re-aligned to the sequences KP715369.1 and KP715368.1 for KLIKK proteases (Ksiazek et al. 2015), using the same filters and parameters as above, as they are poorly assembled in the reference genome used. Coverage was normalized using mean genomic coverage and the length of the interval of interest, and virulence factors were classed as present if >0.5 of the interval was covered and coverage was at least 80% of the expected coverage or if >0.95 of the interval was covered (see supplementary note S1, Supplementary Material online). Comparisons between periods were performed using chi square tests implemented in the stats package in R (R Core Team 2023).

## Supplementary Material

msae017_Supplementary_Data

## Data Availability

FASTQ files with host reads removed and BAM files aligned to *S. mutans*, *T. forsythia*, and *Tr. denticola* are available from the ENA (accession PRJEB64128). Bioinformatic scripts are provided in an external repository hosted on github (https://github.com/iseultj/killuragh_analysis_paper) and archived with Zenodo under DOI 10.5281/zenodo.10535406. Intermediate analysis files are also archived here: https://doi.org/10.5281/zenodo.10024810.
