# Supplementary material for: Ancient Genomes From Bronze Age Remains Reveal Deep Diversity and Recent Adaptive Episodes for Human Oral Pathobionts
Source: Mol Biol Evol. 2024 Mar 27;41(3):msae017. doi: 10.1093/molbev/msae017 (PMC10966897; doi:10.1093/molbev/msae017)
Supplement: msae017_Supplementary_Data [file msae017_supplementary_data.zip › supplement.pdf]

Supplementary Information

Supplementary Note 1: Extended Materials & Methods **1**
Processing 1
Sampling and Sequencing 1
Sequence Data Processing 2
Metagenomic Analysis 2
Taxonomic Assignment 2
Microbiome Preservation 2
Compositional Analysis 3
Metagenomic Assembly 3
Streptococcus mutans Analysis 3
Pangenome Analysis 3
Functional Analysis 3
Mutacin Simulation 3
Phylogenetic Analysis 4
Reference Genome Alignment 5
Modern data processing 6
SNP Ascertainment and Calling 6
Heteroplasmy 6
Killuragh genome identity 7
Tannerella forsythia Analysis 7
Phylogenetic Analysis 7
Virulence Factor Analysis 8
Supplementary Note 2: Archaeological Context **9**
Supplementary Note 3: Metagenome Assembly **9**
Supplementary Note 4: Genotype Concordance for T. forsythia and T. denticola **10**
Supplementary Note 5: Heterozygosity in Ancient Oral Genomes **10**
Supplementary Note 6: Singleton Sites in Divergent T. forsythia Genomes **12**
Supplementary Note 7: Mutacin Branch Lengths and Simulations **12**
Supplementary Figures **13**
References **24**

Supplementary Note 1: Extended Materials & Methods

Processing

Sampling and Sequencing

Prior to powderisation, we mechanically cleaned the surfaces of both teeth and exposed both sides to 30 minutes of UV radiation. For each tooth, we removed as much pulp as possible prior to sampling a section of root (KGH1-A, KGH2-B) and crown (KGH1-E, KGH2-F). KGH1-A, KGH2-B and KGH2-F were subject to the same extraction protocol. Between 75-160mg of tooth powder was incubated in an extraction buffer (20µl 1M Tris-HCL; 17µl SDS 2%; 940µl 0.5M EDTA; 13µl Proteinase K) for 24 hours at 37°C. Following this, the supernatant was collected after centrifugation for 10 minutes at 13,3000rpm. These steps were repeated two more times, resulting in three supernatants, which were then purified using Amicon filters and a 1X Tris-EDTA wash buffer. The protocol for KGH1-E differed slightly, in that the first two incubations were in an EDTA solution and only lasted 30 minutes. Purified extracts were treated with USER enzyme (3 hours for KGH1-A, KGH2-B,F; 1 hour for KGH1-E) and Illumina libraries were created as described in Cassidy et al. (2020), following the Meyer and Kircher (2010) protocol. Multiple aliquots of each library were then amplified using Accuprime Pfx Supermix (Life Technology).

KGH1-A, KGH2-B and KGH2-F were screened at a low sequencing depth on an Illumina MiSeq platform (TrinSeq, Trinity College Dublin) with 65bp single-end sequencing, and USER-treated libraries sequenced to a higher read depth on an Illumina HiSeq 2500 (Macrogen, 100bp single end). USER-treated libraries were used for analysis, with damage patterns from non-USER treated libraries used to confirm the authenticity of this data. A USER-treated library from KGH1-E was screened on an Illumina NovaSeq 6000 with 150bp paired end sequencing (Biosource, UK), and sequenced to higher read depth on an Illumina NovaSeq 6000 (Trinseq, Trinity College Dublin) with 50bp and 100bp paired-end sequencing. See **Table S9** for alignment statistics for each PCR to each taxon discussed here.

Sequence Data Processing

Raw FASTQ files were trimmed to remove residual adapters from sequencing: single end reads were trimmed using cutadapt v1.2.1 with the parameters
"-a AGATCGGAAGAGCACACGTCTGAACTCCAGTCAC -O1 -m 25 -q 25"
and paired end reads were trimmed using AdapterRemoval v2.2.2 with parameters
"--collapse --minadapteroverlap 1 --adapter1 AGATCGGAAGAGCACACGTCTGAACTCCAGTCAC --adapter2 AGATCGGAAGAGCGTCGTGTAGGGAAAGAGTGTACTATTA --minlength 25 --minquality 25 --trimns"

(Martin 2011; Schubert et al. 2016). Read pairs with more than 11 base pairs overlapping were collapsed and these collapsed reads were used for analysis.

Trimmed reads were aligned to hs37d5 using bwa aln with relaxed parameters (-l 165000 -n 0.01 -o 2) (Li and Durbin 2009), and the unaligned reads were converted to FASTQ format for metagenomic analysis. FASTQ files were filtered for exact index matches, deduplicated using prinseq++ with default parameters (Cantu et al. 2019), and were merged to sample level.

Published FASTQ files were downloaded from the ENA, trimmed for adapters as above if necessary and then processed in an identical fashion, excluding the index-matching step.

Metagenomic Analysis

Taxonomic Assignment

Merged FASTQ files were profiled using the tool kraken2 (Wood et al. 2019), using the NCBI Refseq database for bacteria, archaea and viruses (downloaded March 2020). These assignments were then refined using the tool Bracken, with the parameters "-r 65 -t 100" (Lu et al. 2017). OTU tables were then generated from the bracken report files using the tool kraken-biom (Dabdoub 2016).

For species of interest (i.e. red complex bacteria, *S. mutans* and *F. nucleatum*), taxa were identified as "present" in our sequencing data if there were > 10,000 alignable reads, and the edit distance was declining ($-\Delta\% > 0.9$) (see Hübler et al. (2019) for calculation).

Microbiome Preservation

Oral microbiome preservation was assessed by comparing the ancient taxonomic assignments to a comparative dataset of modern oral, skin, gut and soil microbiomes, as well as lab control sequencing data (Human Microbiome Project Consortium 2012; Salter et al. 2014; Johnston et al. 2016; Lloyd-Price et al. 2017). SourceTracker2 was used to estimate the proportion of each sample attributable to each of these potential sources from species-level OTU tables, using a rarefaction depth of 2000 for both source and sink data, due to variable sequencing depths in the comparative data (Knights et al. 2011).

Compositional Analysis

In order to minimise the impact of spurious taxonomic assignment to low-abundance taxa, all species with less than 0.03% total reads from a sample assigned to them were removed from the OTU table.

The taxonomic composition of this data was visualised: first at the genus level (**Figure S2A**), then at the species level within the genus *Streptococcus* (**Figure S2B**). Relative abundance of oral pathobionts was normalised to the sample with the highest abundance of each taxon, and is presented in **Figure 1**.

Authentication

In order to assess the authenticity of the *S. mutans* and *T. forsythia* signals in the teeth from Killuragh, competitive mapping (using the same parameters as above) against representative available genomes for members of the same genus (in the case of *S. mutans*) or family (in the case of *T. forsythia*) was used to confirm that the species identification was correct (**Figure S3,4,5**) (Guellil 2021), while mapDamage analysis on non-UDG treated libraries was used to confirm that the bacterial genomes represented true ancient DNA (**Figure S6**), and were not from more recent contaminants.

Metagenomic Assembly

De novo genome reconstruction was attempted for the metagenomic reads from Killuragh. MEGAHIT was used to assemble contigs from deduplicated, filtered fastq files (see above for processing) (Li et al. 2015). These contigs were then prepared for binning by cutting the assembled contigs into shorter contigs with a maximum length of 10kb, and the initial reads were mapped back to the contigs to calculate the depth of coverage. The contigs were binned using CONCOCT (Alneberg et al. 2014), and the bins were assessed using CheckM (Parks et al. 2015); Taxonomic assignment was carried out using GTDB-Tk (Chaumeil et al. 2019).

***Streptococcus mutans* Analysis**

Pangenome Analysis

A high-quality bin for *S. mutans* was recovered from KGH2-B: this genome was then analysed in the context of modern streptococcus mutans genomes from NCBI's genbank (see **Table S6** for full list of genomes analysed). Genomes were annotated using PROKKA 1.14.6, using the coding sequences annotated in the UA159 assembly as a training set (Ajdić et al. 2002; Seemann 2014). Any duplicate assemblies were removed, and the pangenome pipeline roary v.3.12.0 was used to calculate the pan-genome, with non-default parameters "-e --mafft" to create a core genome alignment and a fast tree with mafft (Page et al. 2015).

Functional Analysis

Mutacin sequences in the pangenome were annotated based on comparison of predicted protein sequence with mutacin sequences deposited in NCBI using diamond blastx (Buchfink et al. 2021), as in Watanabe et al. (2021).

Mutacin Simulation

We reconstructed a 0.4X *S. mutans* genome from a French Neolithic tooth labelled 1H13 (Seguin-Orlando et al. 2021). No reads covering mutacin sequences were detected for this sample. To assess the likelihood of this occurring if mutacin genes were indeed present in 1H13 we carried out simulations.

For each combination of mutacins present in the modern genomes, a modern genome containing that combination was chosen to be simulated as a low-coverage ancient *S. mutans* genome using the tool gargammel (Renaud et al. 2017), using the inferred base misincorporation and fragment length distribution of 1H13. 1000 simulations were run for each mutacin profile, with the command:

```
gargammel -mapdamage results_1H13.UA159.sorted.duprm.grouped/misincorporation.txt double -f
length_frequencies.tab -fa AGATCGGAAGAGCACACGTCTGAACTCCAGTCAC -sa
AGATCGGAAGAGCGTCGTGTAGGGAAAGAGTGTACTATTA --comp 0,0,1 -c 0.4 -rl 101 -o
output/${profile}_${i}/ ${profile}/
```

Here ${i} is the simulation number, ${profile} is the mutacin profile, -c is the coverage, -fa and -sa were the adapters used, -f is the fragment length frequencies from 1H13, –comp simulates just endogenous reads (in this case, *S. mutans* reads) and -rl 101 is the length of the simulated Illumina reads. These simulated reads were then aligned to the *S. mutans* pangenome, as described in the below section on reference genome alignment, without mapping quality thresholds due to closely related homologs in the pangenome. A relaxed approach to detection was taken: if at least one read aligned to any mutacin sequences simulated, a mutacin was considered detected. Results are presented in Supplementary Note 7.

Phylogenetic Analysis

<u>Maximum Likelihood trees</u>

Multiple sequence alignment on the modern and ancient assemblies was performed using the tool SKA (Split Kmer Analysis) with the UA159 sequence used as a reference (Harris 2018). As *S. mutans* undergoes extensive recombination, the tool gubbins was then used to filter out recombining regions prior to phylogenetic analysis (Croucher et al. 2015). The non-default parameter –remove-identical-sequences was used to remove any duplicate sequences.

SNP sites were extracted from this alignment of non-recombining sequences using the tool snp-sites (Page et al. 2016). SNPs were filtered to remove multiallelic sites , as well as sites with > 5% missingness, leaving a total of 7629 sites for analysis.

These sites were used to create a maximum likelihood tree using IQTree (Kalyaanamoorthy et al. 2017; Hoang et al. 2018; Minh et al. 2020). The parameters -m MFP (to select the best-fitting model for sequence evolution) –date and -date-ci 100 were used to incorporate the dating information from the ancient genome in the analysis. 1000 rapid bootstraps were performed to assess tree support. The best-fitting model according to AIC and BIC was GTR+F+ASC+R5, and this was used for maximum likelihood analysis.

<u>Bayesian analysis: BEAST</u>

The same SNP sites were used in a Bayesian phylogenetic analysis using BEAST v.2.6.7 (Bouckaert et al. 2019). The masked alignment from gubbins was filtered for monomorphic sites using the tool snp-sites with the flag -b (Page et al. 2016). The frequency of each nucleotide in this sequence was used to specify invariant sites in the BEAST XML. BEAST Model Test was used to define the site model used, and a relaxed log normal clock was used for the clock model (Drummond et al. 2006; Bouckaert and Drummond 2017). A coalescent bayesian skyline prior was used (Drummond et al. 2005). Two independent runs with 500,000,000 chains and 1,000,000 burnin steps converged for all statistics. ESS was above 200 for nearly all statistics in both runs except for BMT ProportionInvariableSites (ESS ~ 50 in both runs) and ucldMean and rate.mean and rate.variance in one of the runs (ESS 140-150 for all three of these estimates). Therefore, the log files and tree files for these runs were combined using LogCombiner, and the maximum clade credibility tree was calculated using TreeAnnotator from median heights, removing burn-in trees from the first 10 million chains.

<u>Geographic Structure</u>

For each pair of genomes in the phylogenetic tree, their most recent common ancestor (MRCA) was calculated using the R package TreeTools (Smith and Wickham 2023). For each sample, geographic location was encoded as the longitude and latitude of the capital city of the country reported as the isolation source. Distance between geographic locations was calculated using the function distm from the package geosphere (Hijmans 2022). Correlations between geographic distance and internal distance in the tree were calculated using cor.test() in the stats package in R across different time bins (R Core Team 2023).

Reference Genome Alignment

Trimmed FASTQ files were aligned to appropriate reference genomes (**Table S8**) using bwa aln with relaxed parameters (-l 165000 -n 0.01 -o 2) (Li and Durbin 2009). Duplicate reads were removed using picard MarkDuplicates. BAM files were filtered for mapping quality > 25 using samtools and read length > 34 base pairs (Li et al. 2009), and the quality score of the first and last two base pairs of each read were reduced to 0 (“soft clipped”) to mitigate the

impact of post-mortem damage. This was increased to 5bp for non-UDG treated samples. Depth and breadth of coverage was calculated using qualimap v2.2.1 (Okonechnikov et al. 2015). Edit distance distribution was plotted and a summary of the shape of this distribution ("negative distance proportion": $-\Delta\%$) was calculated as in the HOPS pipeline (Hübler et al. 2019). Alignment statistics for new libraries reported here are presented in **Table S9** and **S10**; Summary alignment statistics for published data are presented in **Table S11** (*T. forsythia*) and **Table S12** (*T. denticola*).

Modern data processing

Modern *T. forsythia* genome assemblies were downloaded from Genbank. In order to make these genomes comparable with the ancient genomes aligned to the 92A2 reference (GenBank accession: GCA_000238215.1), pseudo-reads of length 100 nucleotides and a slide of 1 were generated and aligned to the 92A2 reference, as in (Philips et al. 2020). The resulting BAM files were filtered for mapping quality > 25. A similar approach was taken for *Treponema denticola*, but were aligned to the ATCC35405 sequence (GenBank accession: GCA_000008185.1)

SNP Ascertainment and Calling

Modern genomes and ancient genomes above a mean genomic coverage of 2X were used to ascertain SNP sites for use in phylogenetic analyses. Sites were called using GATK's UnifiedGenotyper in discovery mode, using the output mode EMIT_ALL_SITES and a minimum base quality of 30 (McKenna et al. 2010). SNPs were filtered for mean coverage within two standard deviations of the mean genomic coverage, with a hard minimum of 2 reads supporting a genotype call. Any sites flagged as low quality by GATK were also removed from the alignment.

SNPs were called in modern and ancient genomes > 1X using GATK pileup (McKenna et al. 2010). A minimum base quality of 30, mapping quality of 25 and read length of 34 were required. A consensus of at least two reads was required to call a genotype. In order to assess the impact of read depth and mapping quality filters for phylogenetic inference, we assessed the impact of using a minimum read depth of 2-7 and minimum mapping quality 25,30 and 35. The results of this analysis are presented in **Figure S7** (Maximum Likelihood tree comparisons across MQ filters) and **Figure S8** (Read depth filters). SNP recovery at each filtering level is presented in the figure legend. Concordance rates were calculated using plink's –merge-mode 7, with a minimum concordance of 0.995 (OAK005, MQ35 filter).

Heteroplasmy

One way to assess contamination in ancient mammalian genomics is to look at the proportion of heteroplasmic sites in the mitochondrial genome. As bacterial genomes are also haploid, we used this approach to assess strain heterogeneity and contamination by

closely related microbes in our ancient dataset. Briefly, the pileup of variant sites identified in the ascertainment step were filtered for a minimum base quality of 30, and major and minor alleles at each site were identified.

The proportion of heteroplasmic sites both with and without potential post-mortem deamination was calculated, as was the proportion of minor allele calls at these heteroplasmic sites. This was used as an estimate of contamination.

For *T. denticola*, the ability of non-competitive alignment, competitive alignment and metagenomic assembly to distinguish *T. denticola* reads from other treponemal reads was assessed by comparing the number of reads assigned to *T. denticola* and other treponemal species using Kraken2 (Wood et al. 2019). For the assembly, paired end reads with read length 101 were simulated using gargammel from the same fragment length distribution as the KGH1-E alignment to *T. denticola* (Renaud et al. 2017). Simulated reads were trimmed and classified with kraken2 as above. Contigs from the assembly bin were also classified using kraken2, to assess the impact of differing sequence lengths on classification.

Killuragh genome identity

Both *T. forsythia* and *T. denticola* genomes were recovered from two different teeth from the Killuragh individual. Concordance between these genomes was estimated using PLINK –merge-mode 7 for the SNP datasets ascertained for both species (Purcell et al. 2007).

Tannerella forsythia Analysis

Phylogenetic Analysis

Raw distances between analysed sequences were assessed using the function "dna.dist" from the R package ape (Paradis and Schliep 2019).

<u>Maximum Likelihood Trees</u>

Maximum likelihood trees were constructed for a subset of the genome dataset with a maximum per-individual SNP missingness of 40%; this was relaxed to include the African sequence TAF008, the neanderthal-derived sequence GOY005 and KGH2-F, to maximise the temporal depth in the trees and number of usable sites for analysis.

First, the dog-derived *T. forsythia* sequence OH2617_COT023 was used as an outgroup. IQTREE v. 2.2.0.3 was used to reconstruct the tree, using tip dates as prior information (Minh et al. 2020). 1000 rapid bootstraps were used to assess support (Hoang et al. 2018). The sequence model HKY+F+ASC+R2 was chosen by IQTREE's ModelFinder (Kalyaanamoorthy et al. 2017).

GOY005 (the Neanderthal-derived genome) was consistently outgrouping the other genomes, so a tree was constructed without OH2617_COT023, using GOY005 as an outgroup. The same parameters for IQTREE were used, but the model selected by ModelFinder was TVM+F+ASC+R3. When these trees were reconstructed using the HKY+F+ASC+R2 model, an identical topology was recovered.

<u>Expansion Date with BEAST</u>

Because the recent expansion seemed to occur between the medieval and industrial period, all *T. forsythia* sequences dated to the medieval period or more recently were used to investigate the timing of this expansion using BEAST. This was also attempted with all samples used to construct the maximum likelihood tree. However, these BEAST runs did not converge for parameters including tree height, substitution rate and likelihoods. This is likely due to recurrent mutation on long branches, so we restricted our analysis to the Medieval period onwards.

First, the temporal signal in the data was assessed by constructing maximum likelihood trees as above, but without incorporating dating information. TempEst was then used to assess the correlation between tree position and date (Rambaut et al. 2016).

SNP data was filtered for a maximum missingness of 5%, and split into four partitions (first codon position, second codon position, third codon position and non-coding regions). Partitions were defined using the 92A2 genome annotation from RefSeq. These partitions were used in a BEAST analysis using BEAST v2.6.7 in an attempt to date a recent expansion under a Coalescent Bayesian Skyline model (Drummond et al. 2005; Bouckaert et al. 2019). The tree model for all partitions were linked, while the clock and site models were unlinked. Priors for sample ages were set to the median radiocarbon date for dated samples, and to approximate dates for undated samples. The date prior for all modern samples was set to 2015. The site model for all partitions was estimated using bModelTest, and a relaxed lognormal clock prior was used. Invariant sites from the genome were specified for each partition also. Two independent runs of 300 million chains each, with a 3 million pre-burnin and 10% burnin converged with an ESS > 1000 for all statistics with the exception of proportion invariant sites and substitution rate for the 1st codon position partition.

Maximum clade credibility trees were constructed using a 10% burnin and using a minimum 90% posterior using Treeannotator, and Bayesian Skyline plots were constructed using Tracer.

Virulence Factor Analysis

Average coverage across *T. forsythia* virulence factors in each modern and ancient genome was estimated using bedtools genomecov. The coordinates of these virulence factors in the

92A2 reference genome were obtained from Philips et al. (2020). As KLIKK proteases are poorly assembled in the 92A2 reference genome, the ancient reads and modern pseudo-reads were realigned to the sequences KP715369.1 and KP715368.1 (Ksiazek et al. 2015), using the same filters and parameters as above.

As there were large differences in genomic coverage across samples, we normalised the number of reads overlapping the interval of interest by the expected number of reads aligning to the interval of interest.

We then classified a virulence factor as “present” if the normalised reads were greater than 0.8, and the fraction of the interval covered was greater than 0.5 or if more than 95% of the region of interest was covered. As a sanity check, the number of virulence factors classified as “present” were plotted against the mean genomic coverage: there was no relationship between coverage and number of virulence factors.

In order to assess the significance in presence and absence across different categories of virulence factors, chi-square tests were performed (implemented in R’s stats package), comparing presence in pre-industrial compared to industrial and modern samples.

Supplementary Note 2: Archaeological Context

Killuragh cave is one of several small natural limestone caves that penetrate a bluff overlooking the floodplain of the River Mulkear, a tributary of the River Shannon (Woodman et al. 2017). Archaeological excavations in 1993 (Director: J. O'Shaughnessy; Licence: 93E0175) and 1996 (Director: P. Woodman; Licence: 93E0175) led to the recovery of disarticulated human bones, faunal remains and artefacts dating from the Early Mesolithic through to the Late Bronze Age. Stratigraphy in the cave was severely disturbed and dating by association was not possible. Two mandible fragments (KGH1 and KGH2) found in different parts of the cave chamber were radiocarbon dated in 1997. KGH1 returned an Early Bronze Age date (OxA-6748; 2280-2140 cal BC) and KGH2 a Late Mesolithic date (OxA-6749; 4350-4260 cal BC). We sampled two teeth, one from each of the two mandible fragments. However, upon deep sequencing, kinship analysis revealed both teeth to derive from the same individual (Cassidy et al. forthcoming). The mandible fragments were reexamined and it was concluded that they resembled left and right portions of the same mandible. This individual is genetically most closely related to Early Bronze Age samples from Ireland (Cassidy et al. forthcoming), indicating that the OxA-6748 date is correct. The Early Mesolithic date (OxA-6749) reported in 1997 is problematic, therefore; the dated sample may have been contaminated, or a sample incorrectly associated with the KGH2 mandible may have been submitted for dating.

Early Bronze Age radiocarbon dates on a dog mandible, human neonate/infant (0-3 months old) tibia and human infant (circa 1 year old) scapula indicate that the activities at Killuragh cave during this period relate to funerary practices and/or the ritualistic deposition of disarticulated human bones. The recovery of nine pottery sherds derived from three different Early Bronze Age vase urns supports this interpretation.

Supplementary Note 3: Metagenome Assembly

It was possible to assemble metagenome assemblies for both *S. mutans* and *T. denticola* from the non-human reads from Killuragh, using MEGAHIT for contig reconstruction and CONCOCT for binning (Alneberg et al. 2014; Li et al. 2015). 13 "High-Quality" bins (> 90% completeness; < 5% contamination) were reconstructed, including *S. mutans* (Bin ID: Cluster10_KGH2-B) and *T. denticola* (Bin ID: Cluster24_KGH1-E). Other bins were also assigned to oral taxa using GTDB, including *Actinomyces oris*; *Desulfomicrobium orale* and *Desulfobulbus oralis*. There were also 16 "Medium-Quality" bins reconstructed, which included both oral and environmental taxa, although some could not be assigned to anything more specific than the family or even order-level. These results are presented in **Table S13** .

Supplementary Note 4: Genotype Concordance for *T. forsythia* and *T. denticola*

The concordance between the genomes isolated from KGH1-E and KGH2-F for both *T. forsythia* and *T. denticola* was assessed using genotype concordance for the SNPs called (see **Methods**).

For *T. forsythia,* these SNPs were only 93% concordant, suggesting that these may be different strains inhabiting the same mouth. Because of this, the two genomes were not merged for downstream analyses.

For *T. denticola*, these SNPs had a concordance rate of 77%. This may also be due to different strains of *denticola* in the oral microbiome of this individual; this may also be related to difficulties disentangling closely related treponemal sequences in ancient metagenomes, which is discussed further below.

Supplementary Note 5: Heterozygosity in Ancient Oral Genomes

T. denticola is one of the three members of the "red complex" involved in the development of periodontal disease, along with *T. forsythia* and *P. gingivalis* (Socransky et al. 1998). There are many closely related oral treponemes in the human oral microbiome, which poses a unique challenge for *denticola* genome reconstruction from ancient metagenomic data (Zeng et al. 2021; Honap et al. 2023). Although 38 ancient samples appeared to have sufficient *T. denticola* DNA preservation for genome reconstruction, closer inspection using heterozygosity and competitive alignment, as well as assessing the metagenome assembled genome for contamination with closely-related treponemal sequences, demonstrated that it was not possible to disentangle an unambiguous *T. denticola* genome of sufficient quality for genomic analysis from other treponemal genomes.

Although *T. forsythia,* another member of the red complex involved in periodontal disease, has been analysed in the context of ancient DNA, there have been no *T. denticola* genomes published, despite detection in many ancient oral metagenomes (see **Figure 1**). We hypothesise that this could be due to the sheer diversity of treponemal sequences in the oral cavity. We attempted to quantify the impact of this diversity on our analysis, and to assess possible ways of addressing potential contamination issues caused by multiple closely-related species in the same metagenome.

As bacterial genomes are haploid, we assessed heterozygosity across the genome at variant sites ascertained in modern and ancient genomes above a mean coverage of 2X when aligned to a single reference sequence (Genbank accession: GCA_000008185.1). Heterozygosity with and without correction for potential post-mortem damage were assessed (see **Methods** for details), and the proportion of reads with the minor allele at

heteroplasmic sites were also assessed (**Table S14**). The median heterozygosity estimate was 7.6% (5% without any potential post-mortem damage), and the median proportion of reads supporting a minor allele call at heteroplasmic sites was 33.7% (24.6% without PMD). This suggested substantial contamination with closely related treponemal species, or else multiple different strains of *T. denticola* in each oral microbiome. In order to investigate this, we assessed heterozygosity estimates at the same sites in competitive alignments with chromosome-level treponemal assemblies from GenBank, filtering for just one representative genome for each subspecies. Although this did reduce the overall levels of heterozygosity, the median heterozygosity estimate was still > 3%, and a median of 35% of reads at heteroplasmic sites supported the minor allele call. There was also a correlation between heterozygosity estimates and mean coverage (**Figure S9A**), which is likely due to the low number of sites covered by more than one read in most of the competitive alignments.

In addition, average heterozygosity was estimated across all *T. denticola alignments* in 10kB sliding windows with a 1kB step. Although overall heterozygosity is reduced in the competitive alignment, it appears that the windows with the highest level of heterozygosity still have high heterozygosity in the competitive alignment (**Figure S9B**).

The competitive alignment appears to reduce, but not eliminate, the elevated levels of heterozygosity observed in *T. denticola*. This could suggest a combination of mis-mapping of other oral treponemes to the *denticola* reference sequence (mitigated by competitive alignment), as well as a diverse population of *denticola*-like genomes in these samples, which are not being pulled out by competitive alignment.

In addition, the ability of metagenomic assembly to distinguish *denticola*-like data from closely related treponemes was assessed by classifying contigs in the *denticola* bin using kraken2. 97.55% of the *denticola*-binned contigs were still assigned to *Treponema denticola* in the assembly. This result is very similar to the percentage of non-*denticola* assigned reads which align to the *T. denticola* contig in the competitive alignment (2.43%). In contrast, 9.6% of the reads aligning to *T. denticola* in the non-competitive alignment are not classified as *T. denticola* by kraken2. This suggests that the best way to reliably reconstruct *T. denticola* genomes is either to assemble them using metagenomic assembly or to use competitive mapping. However, when sequencing reads were simulated from the *T. denticola* assembly, 8.5% of the *Treponema*-assigned reads were not specifically assigned to *T. denticola* sequences in the kraken2 database: sequence length has a dramatic impact on classification accuracy. It is also worth noting that the Killuragh aligned genome had considerably lower heterozygosity than would be expected for its coverage (**Figure S9A**), so might already be more appropriate for genome reassembly than most ancient samples.

The proportion of heteroplasmic sites both with and without potential post-mortem deamination was assessed, as was the proportion of minor allele calls at these heteroplasmic sites. The results of this analysis are presented in **Table S15**. All samples had <5% estimated heterozygosity.

Supplementary Note 6: Singleton Sites in Divergent *T. forsythia* Genomes

The oldest sequences in the *T. forsythia* dataset were derived from prehistoric African and Neanderthal dental calculus (Fellows Yates et al. 2021), so were hypothesised to be quite diverged sequences. The number of singleton sites in each sample for all sites & transversions only were assessed across a number of different ascertainment and SNP calling approaches as a sanity check. These were normalised by the percentage of the genome covered by at least two reads, as this is the proportion of the genome for which sites can be called using the consensus approach.

A relaxed SNP calling approach was also tested, where just one read had to cover a site to be called, and if more than one read covered a site, the read used to call the SNP was drawn at random. This was used to try and recover diversity in the GOY005 and TAF008 genomes, which were lower coverage (1.5 and 1.8X mean genomic coverage respectively). Although this approach did not recover an excess of singletons in these genomes, it did recover an excess of singletons in several industrial *T. forsythia* genomes, which were excluded from downstream analysis (**Figure S10**).

Other studies have published ancient *T. forsythia* genomes which were not used in this analysis. Philips et al. (2020) report 2 1000-year old and 1 2000-year old genome, but raw sequencing data (with base quality scores included) were not publicly available. Bravo-Lopez et al. (2020) report genomes from pre-Hispanic and Colonial Mexico, but used a capture-based approach, so these genomes were excluded from our analysis to maximise the portion of the *T. forsythia* genome that could be analysed.

Supplementary Note 7: Mutacin Branch Lengths and Simulations

The observation that mutacin positive strains tended to have significantly shorter branch lengths than mutacin-negative strains (**Figure S19**), as well as the absence of mutacins in both KGH2-B and 1H13, led us to hypothesise that mutacin acquisition might have been a recent event. When similarly low-coverage genomes to 1H13 with mutacin profiles seen in the modern data were simulated using gargammel (Renaud et al. 2017), the recall rate for any simulated genomes with at least two mutacins is at least 70% (**Table S7, Figure S20)**. Because of these observations, we think that it is extremely unlikely that either of these prehistoric genomes had more than one mutacin, although it is much more difficult to state if mutacins were completely absent in 1H13, as the recall rates for individual mutacins

tended to be 50% or less, so this result should be taken with caution. However, the most common mutacin profiles in modern *S. mutans* genomes had higher recall rates (e.g. approximately a third of modern genomes carry mutacins 4 and 5, which has a recall rate of >90% in our simulations) (**Table S7**), suggesting that even if 1H13 did carry mutacins, it would not be a common profile seen today. Another 0.4X genome from an 18th century tooth (LM_309_T) was also tested for the presence of mutacins (Willmann et al. 2018). One read with an edit distance of zero was found to map to mutacin 4, one of the more common mutacins in modern genomes (**Table S7**).

Supplementary Figures

Figure S1: SourceTracker2 analysis

Data from the human microbiome project, published lab controls and soil metagenomes were used as potential sources. Each bar corresponds to an individual sample, and samples are grouped by publication and substrate type. Killuragh samples are ordered KGH1-A, KGH1-E, KGH2-B, KGH2-F. Red colours correspond to estimated oral fraction.

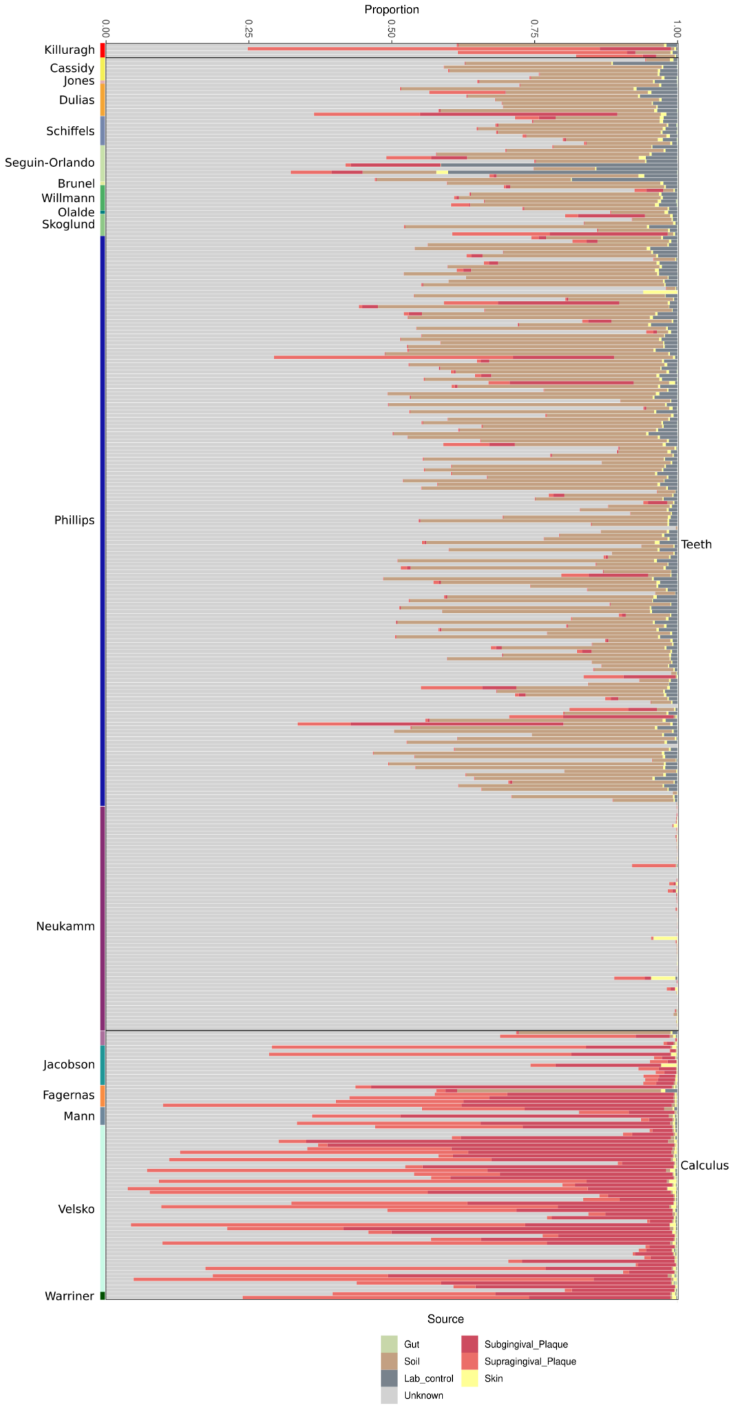

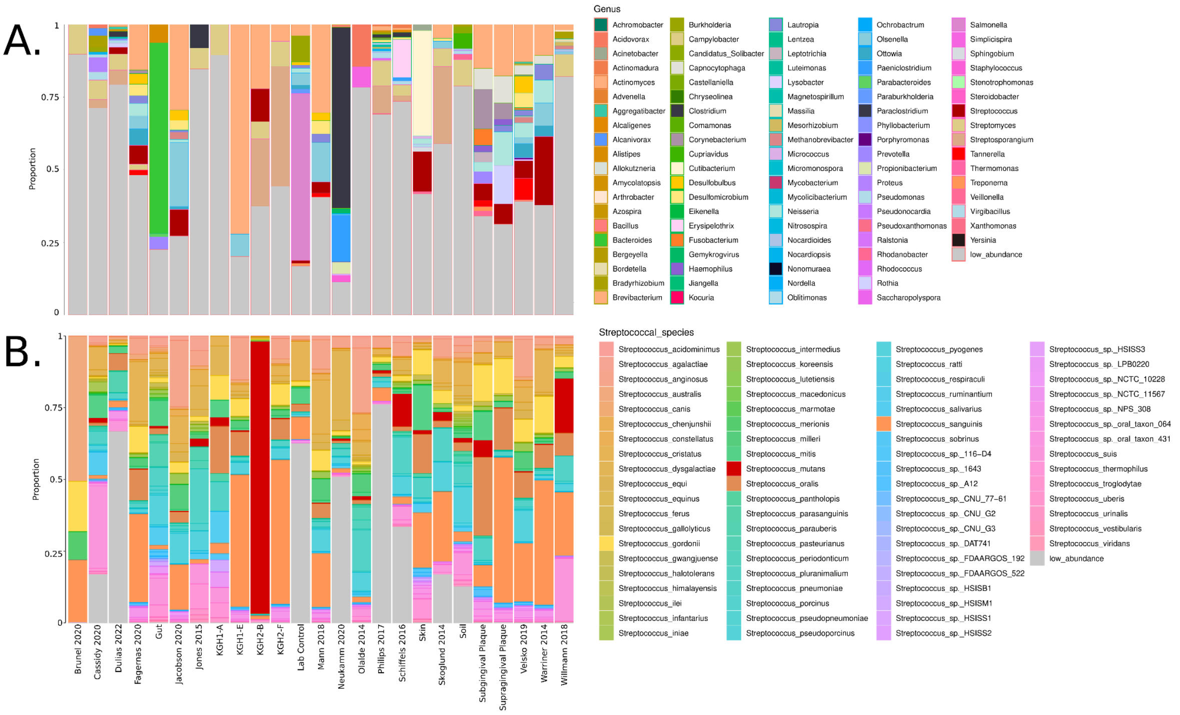

Figure S2. Taxonomic composition

A. Genus composition of each Killuragh aliquot compared to aggregated data from each comparative study. The genus *Streptococcus* is highlighted in dark red. **B.** Composition of streptococcal species assignments in Killuragh aliquots compared to aggregated data from each comparative study. *Streptococcus mutans* is highlighted in red.

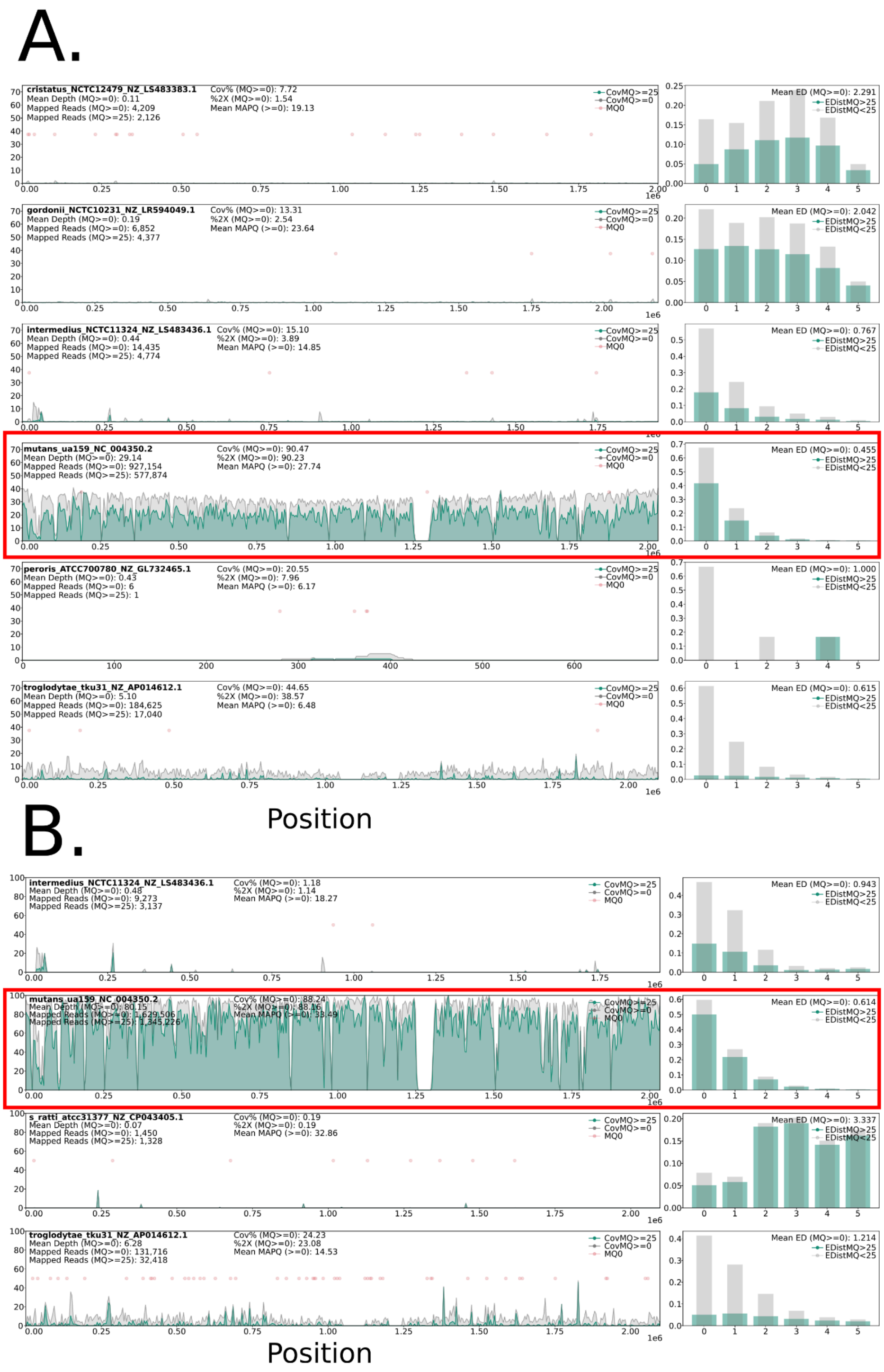

Figure S3. Competitive mapping: Streptococcus

Coverage and edit distance plots for MQ25 (green) and MQ0 BAM files aligned to competitive mapping datasets for Streptococci (A (KGH2-B raw reads), B (S. mutans MAG pseudoreads)). For clarity, only contigs with >= 0.05X mean coverage were plotted. Target reference genomes are highlighted in red, and show high specificity, as well as strictly declining edit distances, supporting the authenticity of our data. Coverage and edit distance plots were generated using Guellil (2021).

and Tannerellaceae (C (KGH1-E raw reads), D (KGH2-F raw reads)).

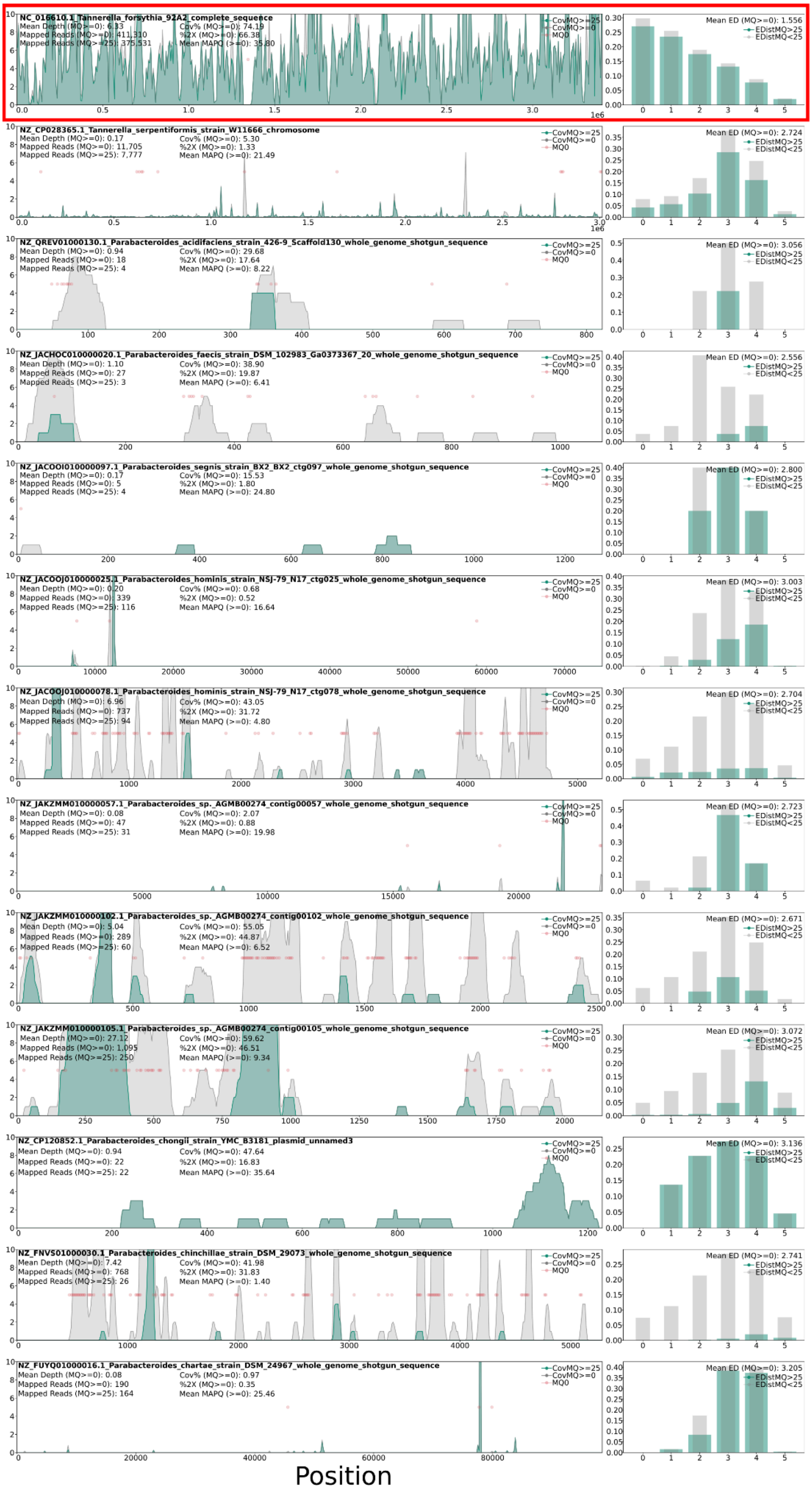
NC_016610.1_Tannerella_forsythia_92A2_complete_sequence
Mean Depth (MQ>=0): 6.33
Mapped Reads (MQ>=0): 411,310
Mapped Reads (MQ>=25): 375,531
Cov% (MQ>=0): 74.19
%2X (MQ>=0): 66.38
Mean MAPQ (>=0): 35.80
Mean ED (MQ>=0): 1.556
NZ_CP028365.1_Tannerella_serpentiformis_strain_W11666_chromosome
Mean Depth (MQ>=0): 0.17
Mapped Reads (MQ>=0): 11,705
Mapped Reads (MQ>=25): 7,777
Cov% (MQ>=0): 5.30
%2X (MQ>=0): 1.33
Mean MAPQ (>=0): 21.49
Mean ED (MQ>=0): 2.724
NZ_QREV01000130.1_Parabacteroides_acidifaciens_strain_426-9_Scaffold130_whole_genome_shotgun_sequence
Mean Depth (MQ>=0): 0.94
Mapped Reads (MQ>=0): 18
Mapped Reads (MQ>=25): 4
Cov% (MQ>=0): 29.68
%2X (MQ>=0): 17.64
Mean MAPQ (>=0): 8.22
Mean ED (MQ>=0): 3.056
NZ_JACHOC010000020.1_Parabacteroides_faecis_strain_DSM_102983_Ga0373367_20_whole_genome_shotgun_sequence
Mean Depth (MQ>=0): 1.10
Mapped Reads (MQ>=0): 27
Mapped Reads (MQ>=25): 3
Cov% (MQ>=0): 38.90
%2X (MQ>=0): 19.87
Mean MAPQ (>=0): 6.41
Mean ED (MQ>=0): 2.556
NZ_JACOOI010000097.1_Parabacteroides_segnis_strain_BX2_BX2_ctg097_whole_genome_shotgun_sequence
Mean Depth (MQ>=0): 0.17
Mapped Reads (MQ>=0): 5
Mapped Reads (MQ>=25): 4
Cov% (MQ>=0): 15.53
%2X (MQ>=0): 1.80
Mean MAPQ (>=0): 24.80
Mean ED (MQ>=0): 2.800
NZ_JACOOJ010000025.1_Parabacteroides_hominis_strain_NSJ-79_N17_ctg025_whole_genome_shotgun_sequence
Mean Depth (MQ>=0): 0.20
Mapped Reads (MQ>=0): 339
Mapped Reads (MQ>=25): 116
Cov% (MQ>=0): 0.68
%2X (MQ>=0): 0.52
Mean MAPQ (>=0): 16.64
Mean ED (MQ>=0): 3.003
NZ_JACOOJ010000078.1_Parabacteroides_hominis_strain_NSJ-79_N17_ctg078_whole_genome_shotgun_sequence
Mean Depth (MQ>=0): 6.96
Mapped Reads (MQ>=0): 737
Mapped Reads (MQ>=25): 94
Cov% (MQ>=0): 43.05
%2X (MQ>=0): 31.72
Mean MAPQ (>=0): 4.80
Mean ED (MQ>=0): 2.704
NZ_JAKZMM010000057.1_Parabacteroides_sp._AGMB00274_contig00057_whole_genome_shotgun_sequence
Mean Depth (MQ>=0): 0.08
Mapped Reads (MQ>=0): 47
Mapped Reads (MQ>=25): 31
Cov% (MQ>=0): 2.07
%2X (MQ>=0): 0.88
Mean MAPQ (>=0): 19.98
Mean ED (MQ>=0): 2.723
NZ_JAKZMM010000102.1_Parabacteroides_sp._AGMB00274_contig00102_whole_genome_shotgun_sequence
Mean Depth (MQ>=0): 5.04
Mapped Reads (MQ>=0): 289
Mapped Reads (MQ>=25): 60
Cov% (MQ>=0): 55.05
%2X (MQ>=0): 44.87
Mean MAPQ (>=0): 6.52
Mean ED (MQ>=0): 2.671
NZ_JAKZMM010000105.1_Parabacteroides_sp._AGMB00274_contig00105_whole_genome_shotgun_sequence
Mean Depth (MQ>=0): 27.12
Mapped Reads (MQ>=0): 1,095
Mapped Reads (MQ>=25): 250
Cov% (MQ>=0): 59.62
%2X (MQ>=0): 46.51
Mean MAPQ (>=0): 9.34
Mean ED (MQ>=0): 3.072
NZ_CP120852.1_Parabacteroides_chongii_strain_YMC_B3181_plasmid_unnamed3
Mean Depth (MQ>=0): 0.94
Mapped Reads (MQ>=0): 22
Mapped Reads (MQ>=25): 22
Cov% (MQ>=0): 47.64
%2X (MQ>=0): 16.83
Mean MAPQ (>=0): 35.64
Mean ED (MQ>=0): 3.136
NZ_FNVS01000030.1_Parabacteroides_chinchillae_strain_DSM_29073_whole_genome_shotgun_sequence
Mean Depth (MQ>=0): 7.42
Mapped Reads (MQ>=0): 768
Mapped Reads (MQ>=25): 26
Cov% (MQ>=0): 41.98
%2X (MQ>=0): 31.83
Mean MAPQ (>=0): 1.40
Mean ED (MQ>=0): 2.741
NZ_FUYQ01000016.1_Parabacteroides_chartae_strain_DSM_24967_whole_genome_shotgun_sequence
Mean Depth (MQ>=0): 0.08
Mapped Reads (MQ>=0): 190
Mapped Reads (MQ>=25): 164
Cov% (MQ>=0): 0.97
%2X (MQ>=0): 0.35
Mean MAPQ (>=0): 25.46
Mean ED (MQ>=0): 3.205
CovMQ>=25
CovMQ>=0
MQ0
EDistMQ>25
EDistMQ<25
Position

Figure S4. Competitive mapping: KGH1-E and Tannerellaceae

Coverage and edit distance plots for MQ25 (green) and MQ0 BAM files aligned to competitive mapping datasets for Tannerellaceae. For clarity, only contigs with >= 0.05X mean coverage were plotted. *T. forsythia* is highlighted in red, and shows high specificity, as well as strictly declining edit distances, supporting the authenticity of our data. Coverage and edit distance plots were generated using Guellil (2021).

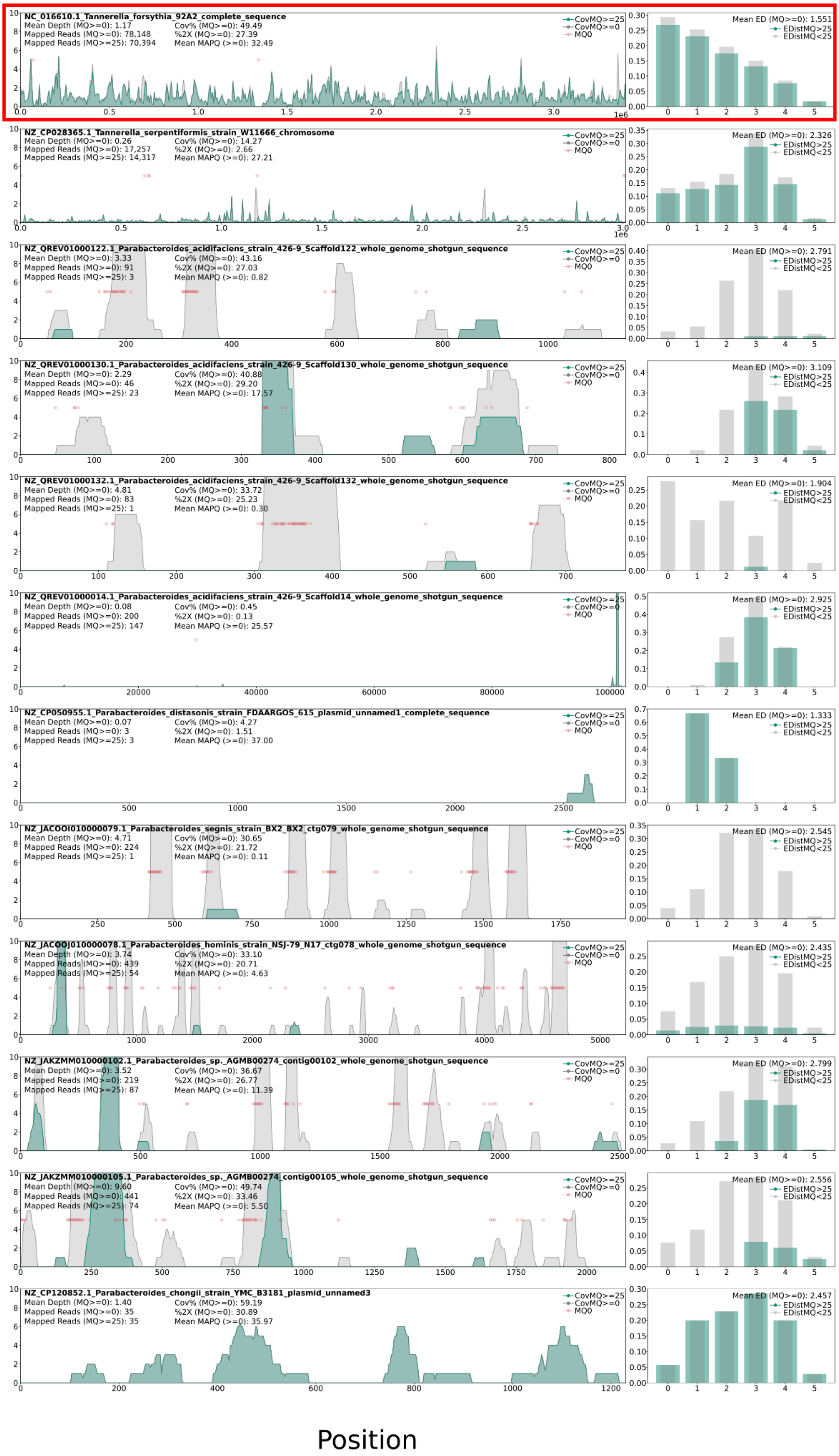
NC_016610.1_Tannerella_forsythia_92A2_complete_sequence
Mean Depth (MQ>=0): 1.17
Mapped Reads (MQ>=0): 78,148
Mapped Reads (MQ>=25): 70,394
Cov% (MQ>=0): 49.49
%2X (MQ>=0): 27.39
Mean MAPQ (>=0): 32.49
Mean ED (MQ>=0): 1.551
NZ_CP028365.1_Tannerella_serpentiformis_strain_W11666_chromosome
Mean Depth (MQ>=0): 0.26
Mapped Reads (MQ>=0): 17,257
Mapped Reads (MQ>=25): 14,317
Cov% (MQ>=0): 14.27
%2X (MQ>=0): 2.66
Mean MAPQ (>=0): 27.21
Mean ED (MQ>=0): 2.326
NZ_QREV01000122.1_Parabacteroides_acidifaciens_strain_426-9_Scaffold122_whole_genome_shotgun_sequence
Mean Depth (MQ>=0): 3.33
Mapped Reads (MQ>=0): 91
Mapped Reads (MQ>=25): 3
Cov% (MQ>=0): 43.16
%2X (MQ>=0): 27.03
Mean MAPQ (>=0): 0.82
Mean ED (MQ>=0): 2.791
NZ_QREV01000130.1_Parabacteroides_acidifaciens_strain_426-9_Scaffold130_whole_genome_shotgun_sequence
Mean Depth (MQ>=0): 2.29
Mapped Reads (MQ>=0): 46
Mapped Reads (MQ>=25): 23
Cov% (MQ>=0): 40.88
%2X (MQ>=0): 29.20
Mean MAPQ (>=0): 17.57
Mean ED (MQ>=0): 3.109
NZ_QREV01000132.1_Parabacteroides_acidifaciens_strain_426-9_Scaffold132_whole_genome_shotgun_sequence
Mean Depth (MQ>=0): 4.81
Mapped Reads (MQ>=0): 83
Mapped Reads (MQ>=25): 1
Cov% (MQ>=0): 33.72
%2X (MQ>=0): 25.23
Mean MAPQ (>=0): 0.30
Mean ED (MQ>=0): 1.904
NZ_QREV01000014.1_Parabacteroides_acidifaciens_strain_426-9_Scaffold14_whole_genome_shotgun_sequence
Mean Depth (MQ>=0): 0.08
Mapped Reads (MQ>=0): 200
Mapped Reads (MQ>=25): 147
Cov% (MQ>=0): 0.45
%2X (MQ>=0): 0.13
Mean MAPQ (>=0): 25.57
Mean ED (MQ>=0): 2.925
NZ_CP050955.1_Parabacteroides_distasonis_strain_FDAARGOS_615_plasmid_unnamed1_complete_sequence
Mean Depth (MQ>=0): 0.07
Mapped Reads (MQ>=0): 3
Mapped Reads (MQ>=25): 3
Cov% (MQ>=0): 4.27
%2X (MQ>=0): 1.51
Mean MAPQ (>=0): 37.00
Mean ED (MQ>=0): 1.333
NZ_JACOOI010000079.1_Parabacteroides_segnis_strain_BX2_BX2_ctg079_whole_genome_shotgun_sequence
Mean Depth (MQ>=0): 4.71
Mapped Reads (MQ>=0): 224
Mapped Reads (MQ>=25): 1
Cov% (MQ>=0): 30.65
%2X (MQ>=0): 21.72
Mean MAPQ (>=0): 0.11
Mean ED (MQ>=0): 2.545
NZ_JACOOJ010000078.1_Parabacteroides_hominis_strain_NSJ-79_N17_ctg078_whole_genome_shotgun_sequence
Mean Depth (MQ>=0): 3.74
Mapped Reads (MQ>=0): 439
Mapped Reads (MQ>=25): 54
Cov% (MQ>=0): 33.10
%2X (MQ>=0): 20.71
Mean MAPQ (>=0): 4.63
Mean ED (MQ>=0): 2.435
NZ_JAKZMM010000102.1_Parabacteroides_sp._AGMB00274_contig00102_whole_genome_shotgun_sequence
Mean Depth (MQ>=0): 3.52
Mapped Reads (MQ>=0): 219
Mapped Reads (MQ>=25): 87
Cov% (MQ>=0): 36.67
%2X (MQ>=0): 26.77
Mean MAPQ (>=0): 11.39
Mean ED (MQ>=0): 2.799
NZ_JAKZMM010000105.1_Parabacteroides_sp._AGMB00274_contig00105_whole_genome_shotgun_sequence
Mean Depth (MQ>=0): 9.60
Mapped Reads (MQ>=0): 441
Mapped Reads (MQ>=25): 74
Cov% (MQ>=0): 49.74
%2X (MQ>=0): 33.46
Mean MAPQ (>=0): 5.50
Mean ED (MQ>=0): 2.556
NZ_CP120852.1_Parabacteroides_chongii_strain_YMC_B3181_plasmid_unnamed3
Mean Depth (MQ>=0): 1.40
Mapped Reads (MQ>=0): 35
Mapped Reads (MQ>=25): 35
Cov% (MQ>=0): 59.19
%2X (MQ>=0): 30.89
Mean MAPQ (>=0): 35.97
Mean ED (MQ>=0): 2.457
CovMQ>=25
CovMQ>=0
MQ0
EDistMQ>25
EDistMQ<25
Position

Figure S5. Competitive mapping: KGH2-F and Tannerellaceae

Coverage and edit distance plots for MQ25 (green) and MQ0 BAM files aligned to competitive mapping datasets for Tannerellaceae. For clarity, only contigs with >= 0.05X mean coverage were plotted. *T. forsythia* is highlighted in red, and shows high specificity, as well as strictly declining edit distances, supporting the authenticity of our data. Coverage and edit distance plots were generated using Guellil (2021).

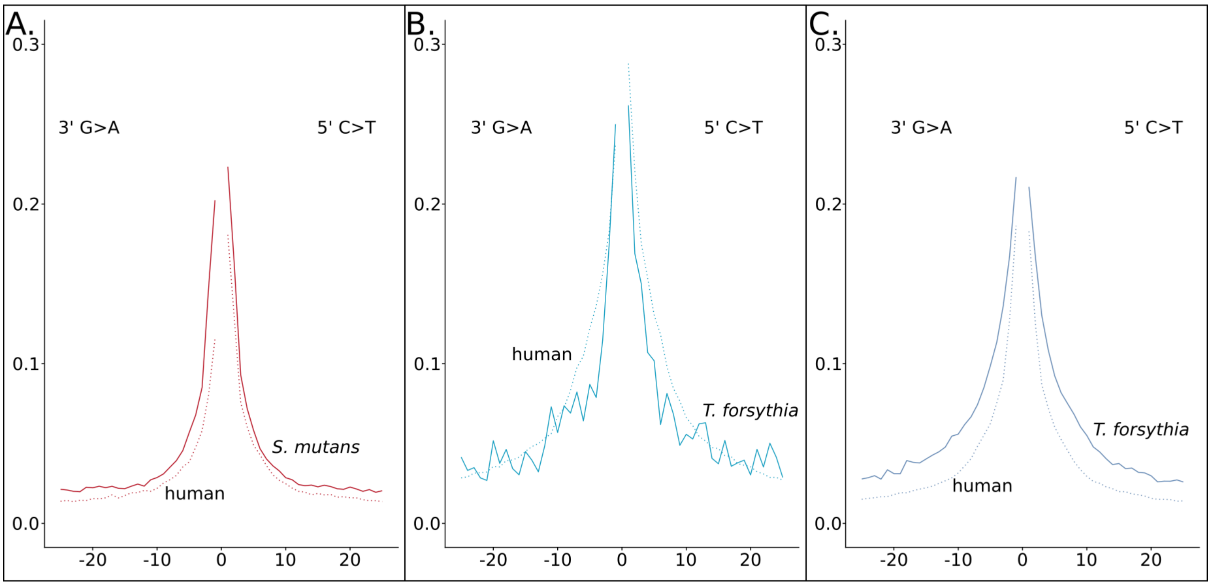

Figure S6. mapDamage for human and bacterial alignments.

Transition rate at 3 and 5’ ends of non-UDG treated reads aligned to human (dotted line) and bacterial (solid line) genomes. **A.** KGH2-B aligned to *S. mutans* **B.** KGH2-F aligned to *T. forsythia* **C.** KGH1-E aligned to *T. forsythia*.

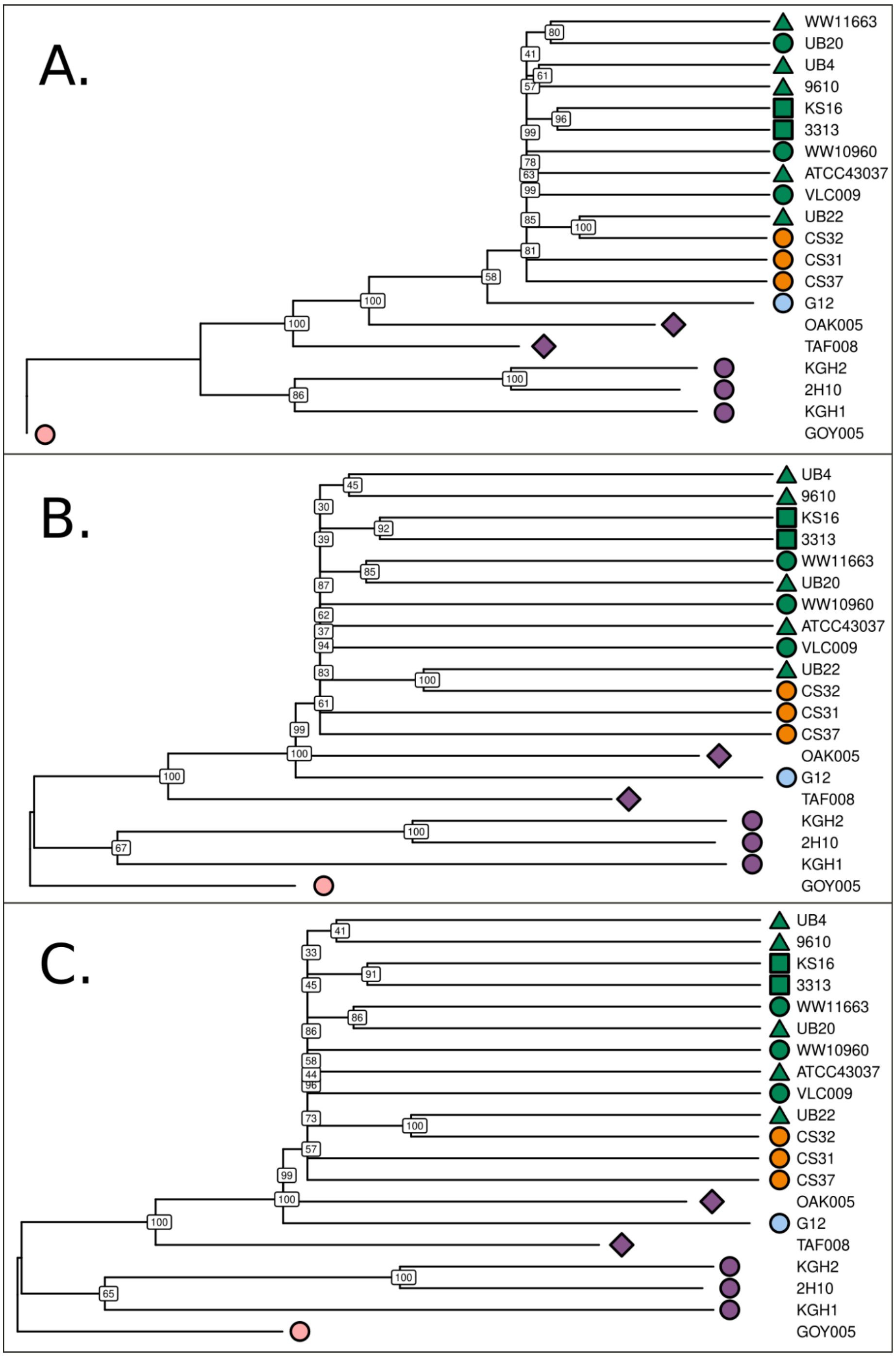

Figure S7. Mapping Quality comparisons

Maximum likelihood trees were constructed with variable mapping quality to assess the impact of different filters on tree topology **A.** Minimum mapping quality 25 (46029 sites; 7179 parsimony-informative); **B.** MQ30 (37350 sites; 4872 parsimony-informative); **C.** MQ35 (37350 sites; 4872 parsimony-informative)

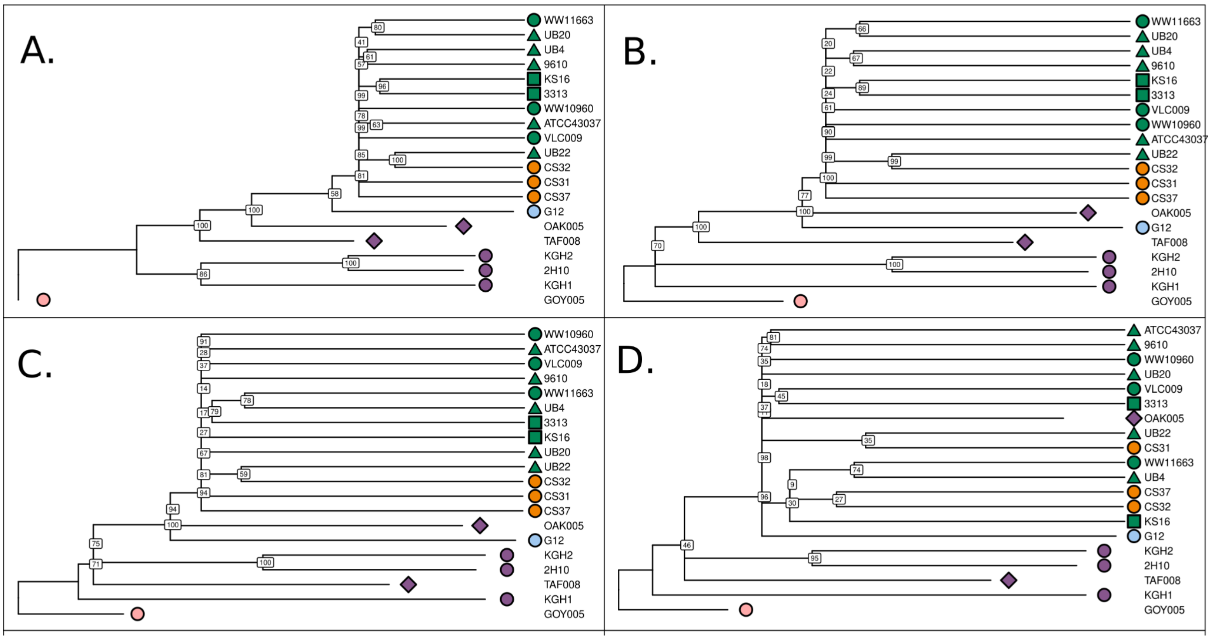

Figure S8. Read Depth comparisons

The impact of minimum read depth for SNP calling was assessed by comparing maximum likelihood tree topologies and support. All variable read depth files were called from BAM files with a minimum mapping quality of 25. **A.** Minimum read depth 2 (46029 sites; 7179 parsimony-informative) **B.** Minimum read depth 3 (16934 sites; 2122 parsimony-informative) **C.** Minimum read depth 4 (4712 sites; 527 parsimony-informative) **D.** Minimum read depth 5 (1285 sites; 137 parsimony-informative).

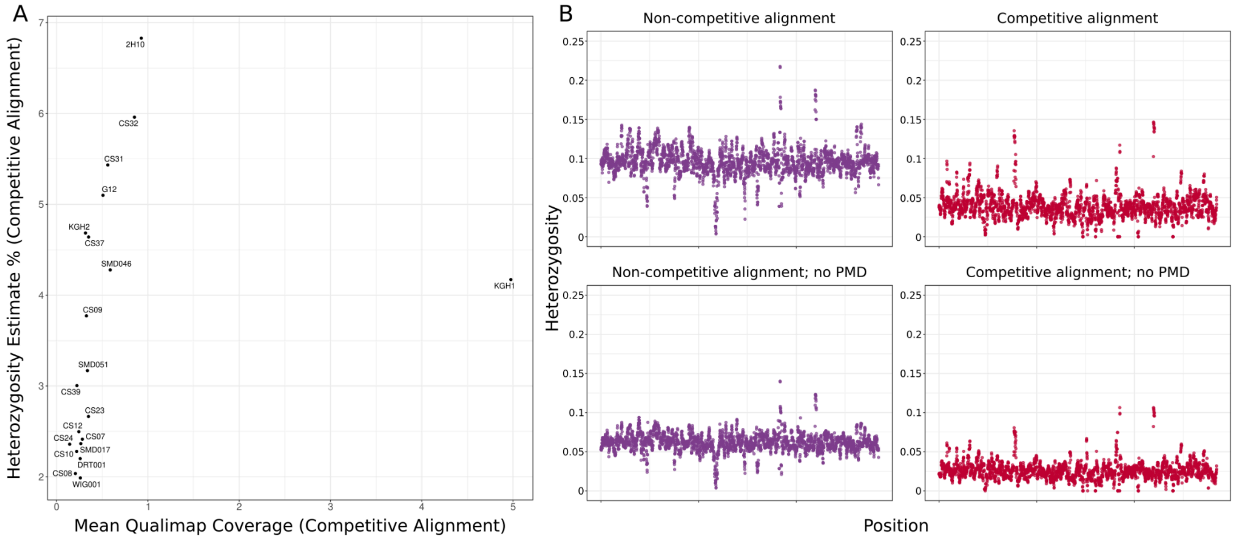

Figure S9: Estimated *T. denticola* heterozygosity

A. Per-sample for competitive alignment. Plotted against average coverage of the *T. denticola* contig in the competitive alignment dataset. **B**. Average heterozygosity across all samples for 10kB windows with 1kB steps. Average heterozygosity is considered for both non-competitive (purple) and competitive (red) alignments, with and without considering possible post-mortem damage (PMD).

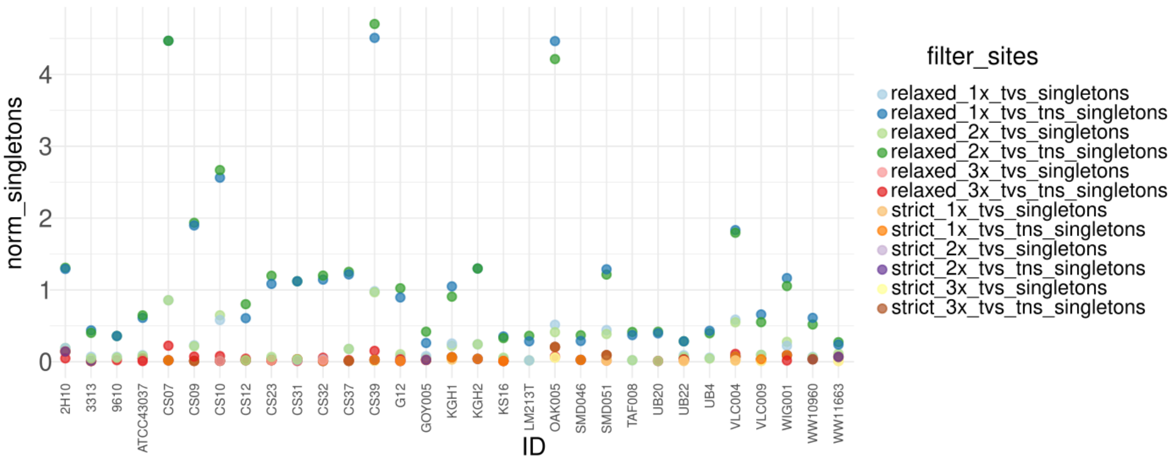

Figure S10. Normalised singleton counts

Number of singletons for each SNP calling approach, normalised by the percentage of the genome covered by at least 2 reads. Relaxed requires just one read covering a site; strict requires at least two reads covering a site.

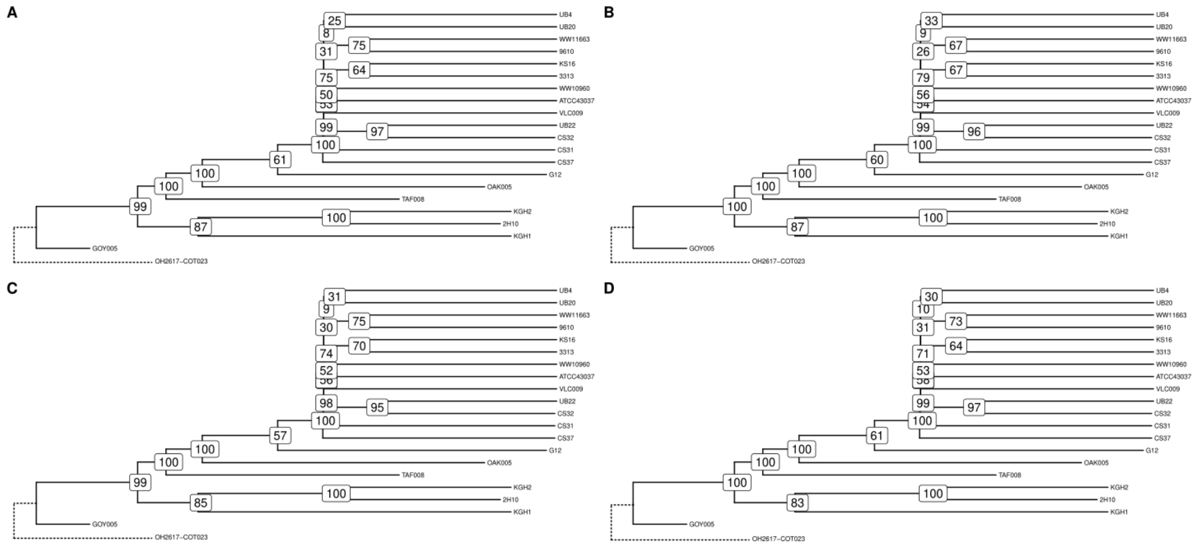

Figure S11: Choice of outgroup.

The dog-derived *T. forsythia* genome OH2167-COT023 was used as an outgroup in exploratory maximum likelihood phylogenetic analyses. GOY005 consistently outgrouped all other hominid-derived *T. forsythia* sequences, so was used as the outgroup in the final phylogenetic analyses.

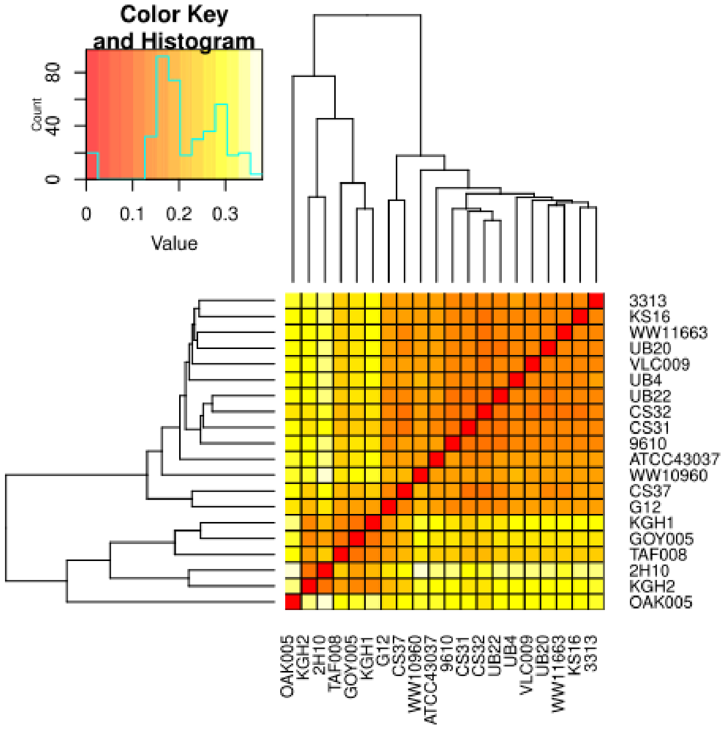

Figure S12. Raw Distances between *T. forsythia* sequences.

The raw distances between the final sequences used for maximum-likelihood tree reconstruction were assessed using the dist.dna function from the R package ape.

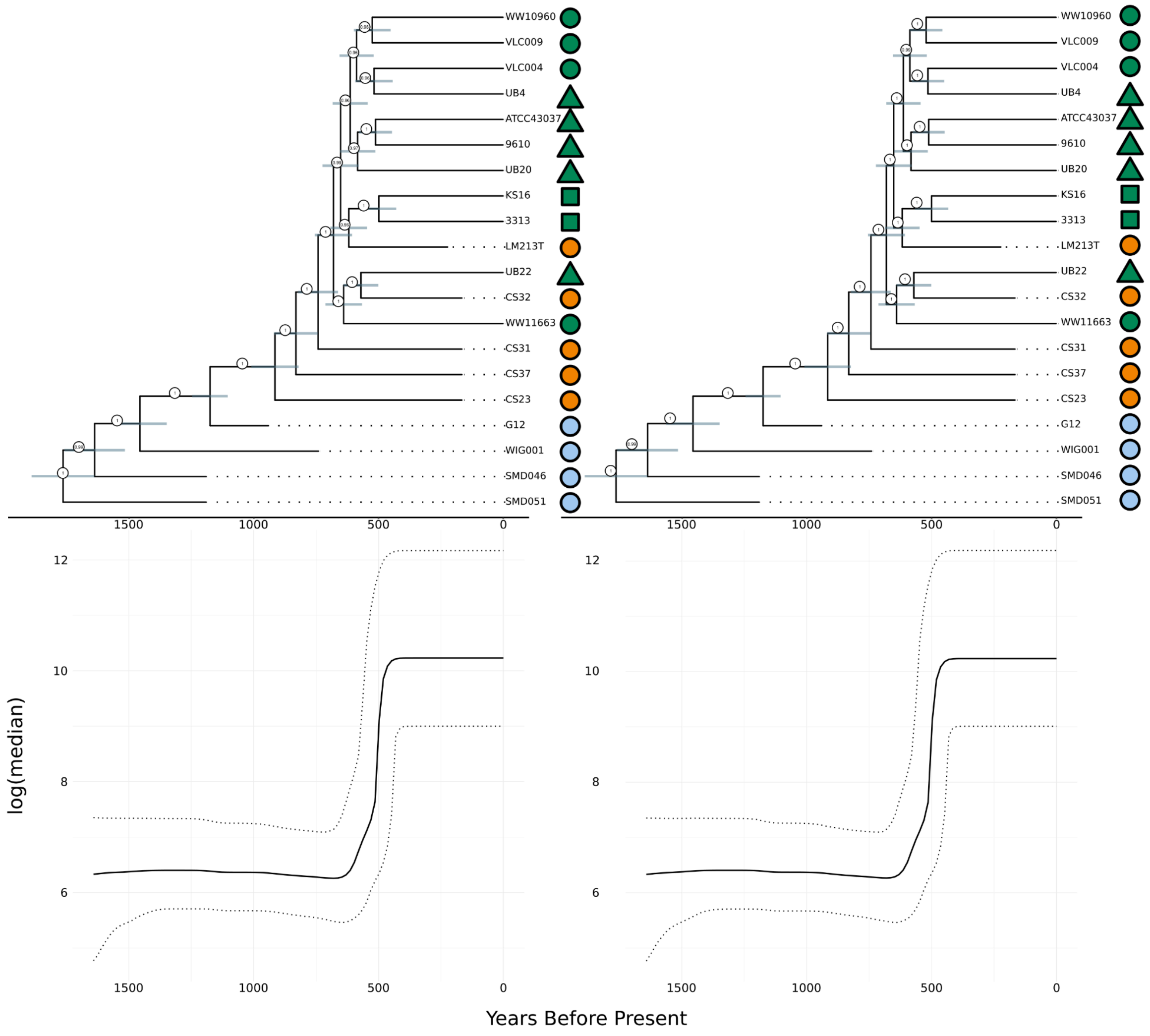

Figure S13. Dating divergence times

Phylogenetic structure and Bayesian skyline plots of *T. forsythia* genomes with a mean genomic coverage above 1X from the medieval period onwards from two independent BEAST runs. Variants were only called if there were a minimum of two reads supporting the call.

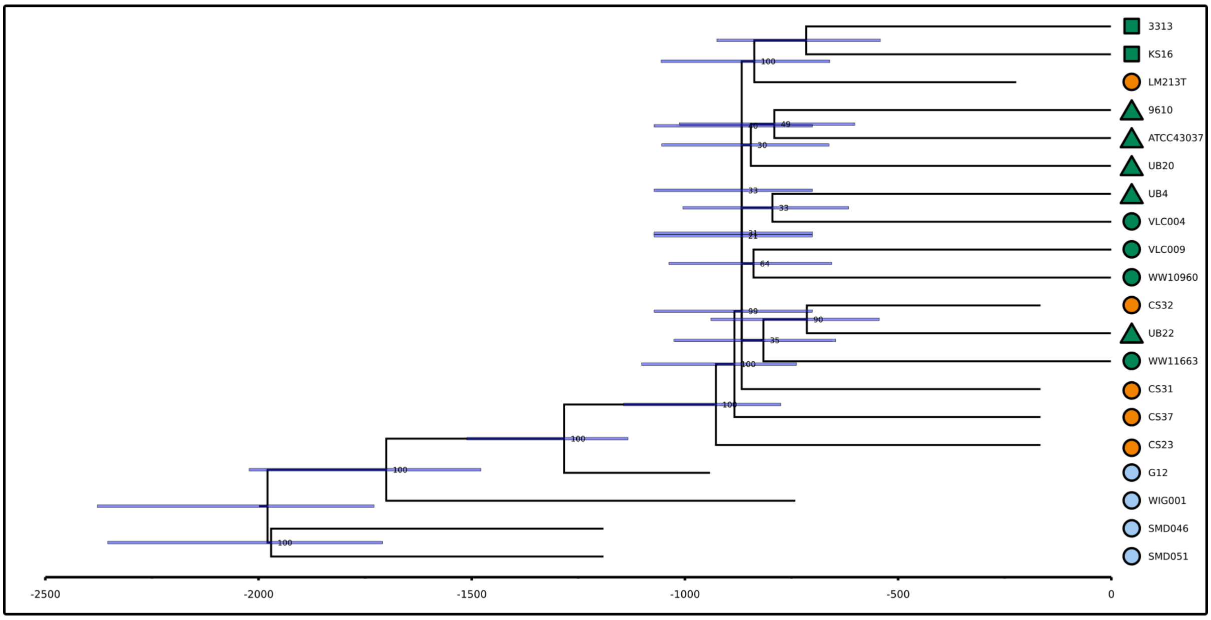

Figure S14. Maximum Likelihood Tree with Medieval Data Onwards

Maximum Likelihood Tree constructed using IQTree with the same dataset as used in the BEAST analysis. Both the topology and timing of the post-medieval expansion broadly agrees across both analyses.

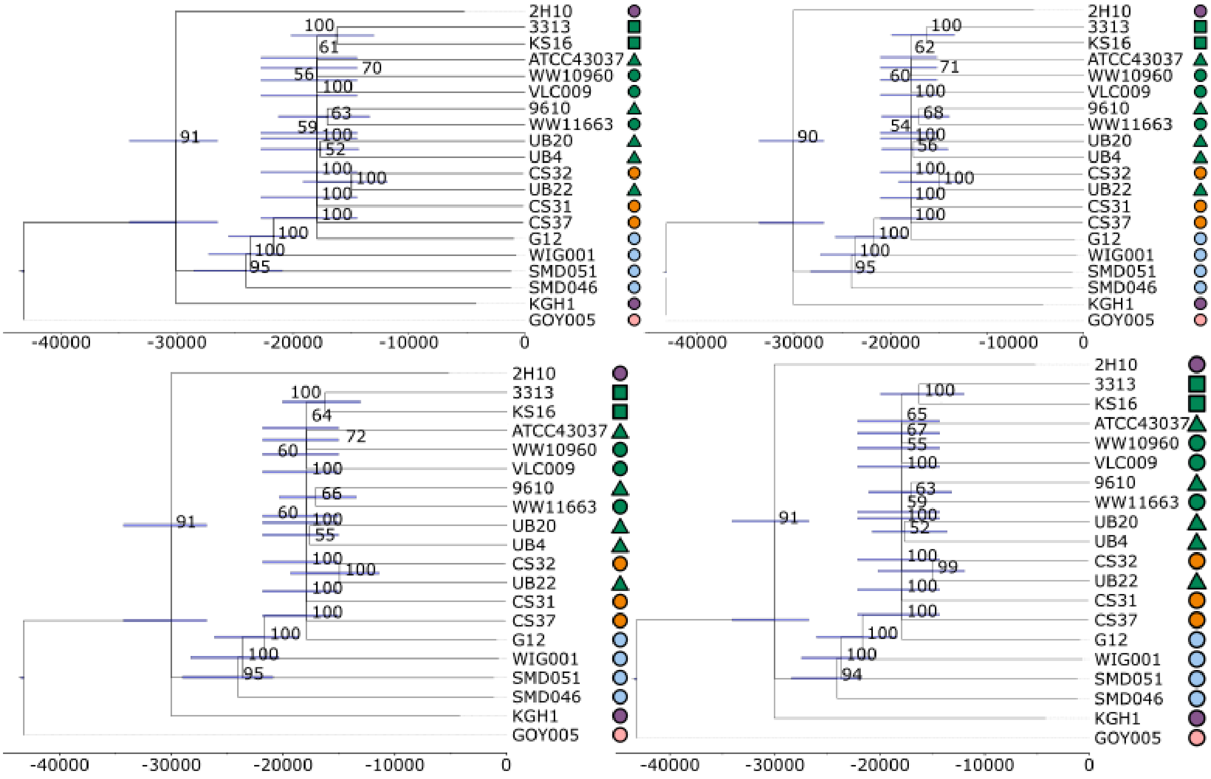

Figure S15. Placement of Medieval individuals in maximum likelihood tree. Maximum Likelihood Tree with Prehistoric, Industrial and modern samples >5X and Medieval

individuals. The order in which 2H10, KGH1 and the branch leading to medieval onwards samples split is not clear, and is represented as a trifurcation.

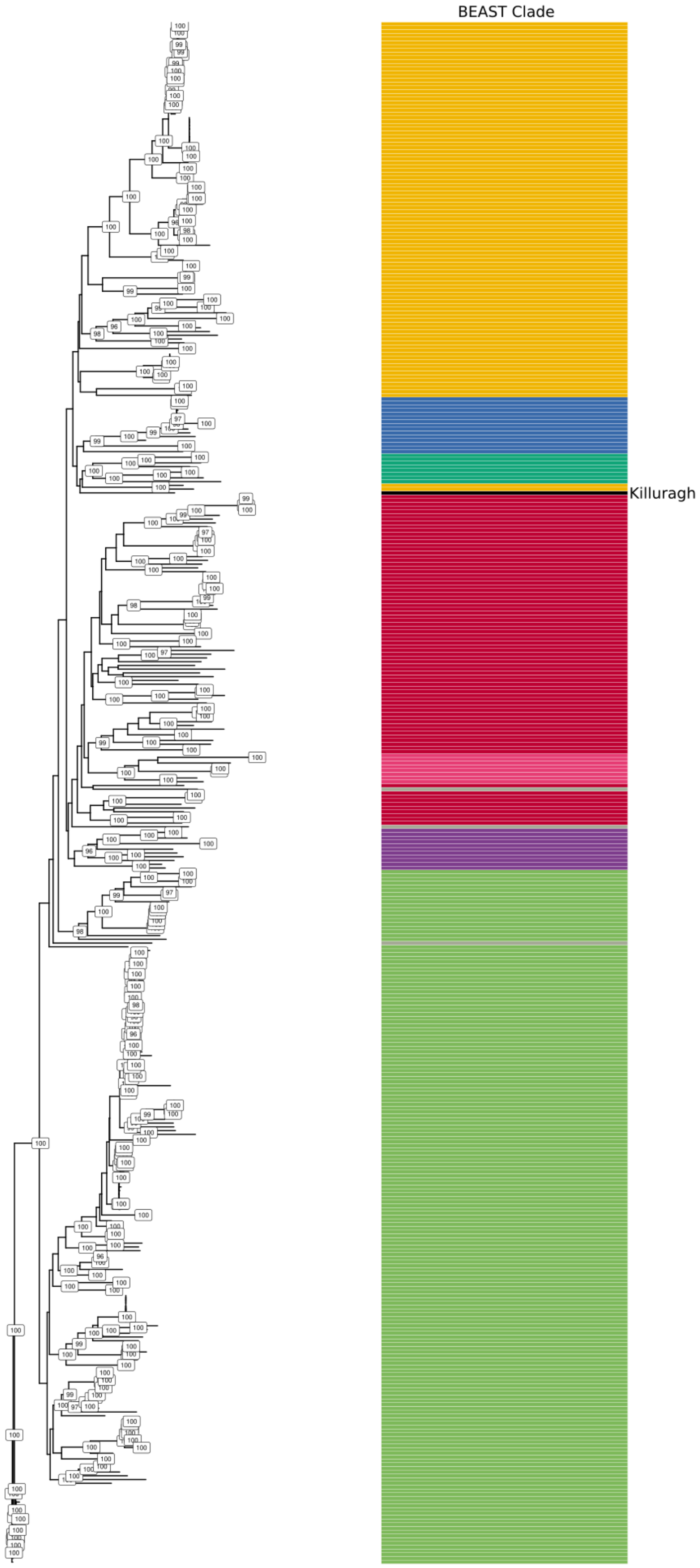

Figure S16. Concordance between Maximum Likelihood tree and BEAST clades.
Colour corresponds to BEAST clades in Figure 3A; Nodes with strong support in the ML tree are labelled with the UFboot values from IQTree.

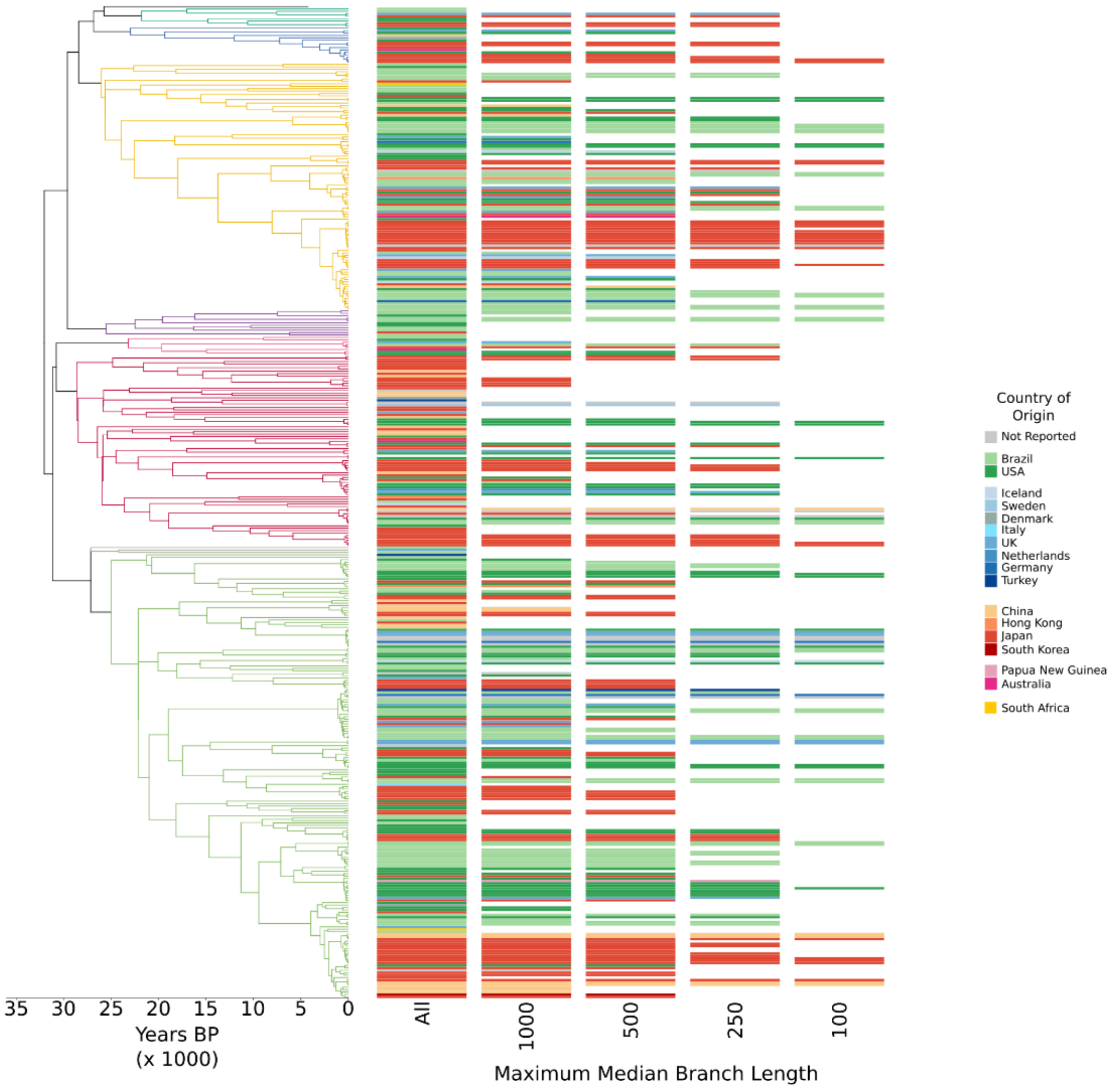

Figure S17. Geographic structure in recent expansions.

Reported countries of origin for all *S. mutans* genomes and those with median estimated branch lengths less than 1000, 500, 250 and 100 were plotted against the estimated BEAST tree to investigate geographic structure. For each pair of genomes in the tree, median height of their most recent common ancestor was plotted against geographic distance between the capital cities of the countries they were isolated in. This was filtered for pairs of samples with a most recent common ancestor more recent than 100 years, 250 years, 500 years, 1000 years and more than 1000 years ago.

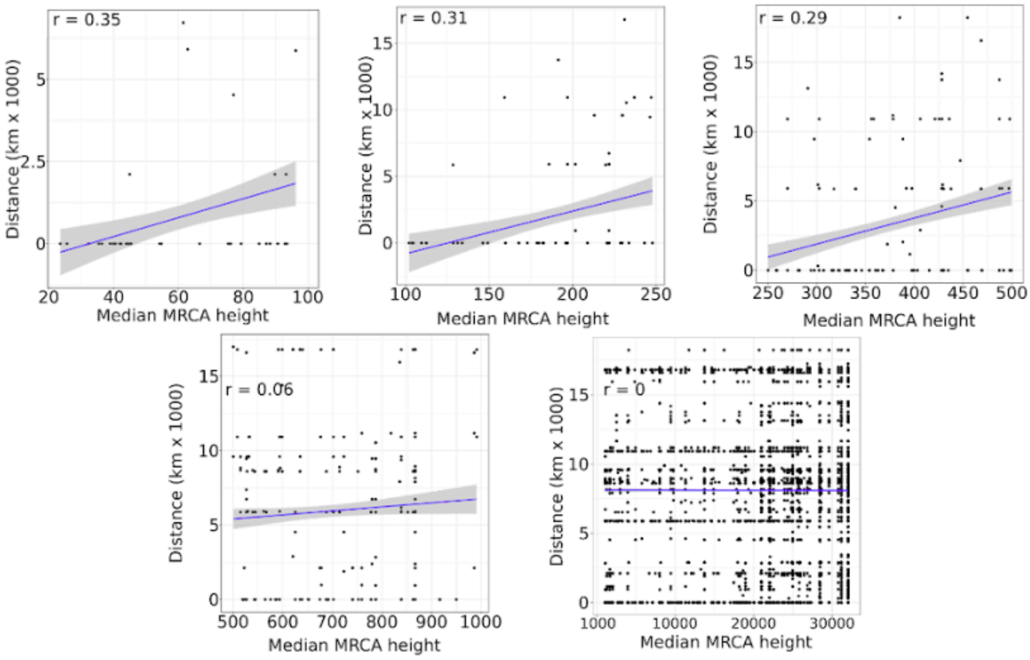

Figure S18. Correlation between geographic distance and median MRCA height

A regression line (plotted using ggplot's geom_smooth function using the method "lm") was plotted to visualise the trend in each time bin. The correlation between phylogenetic distance and geographic distance for each bin is reported on each plot.

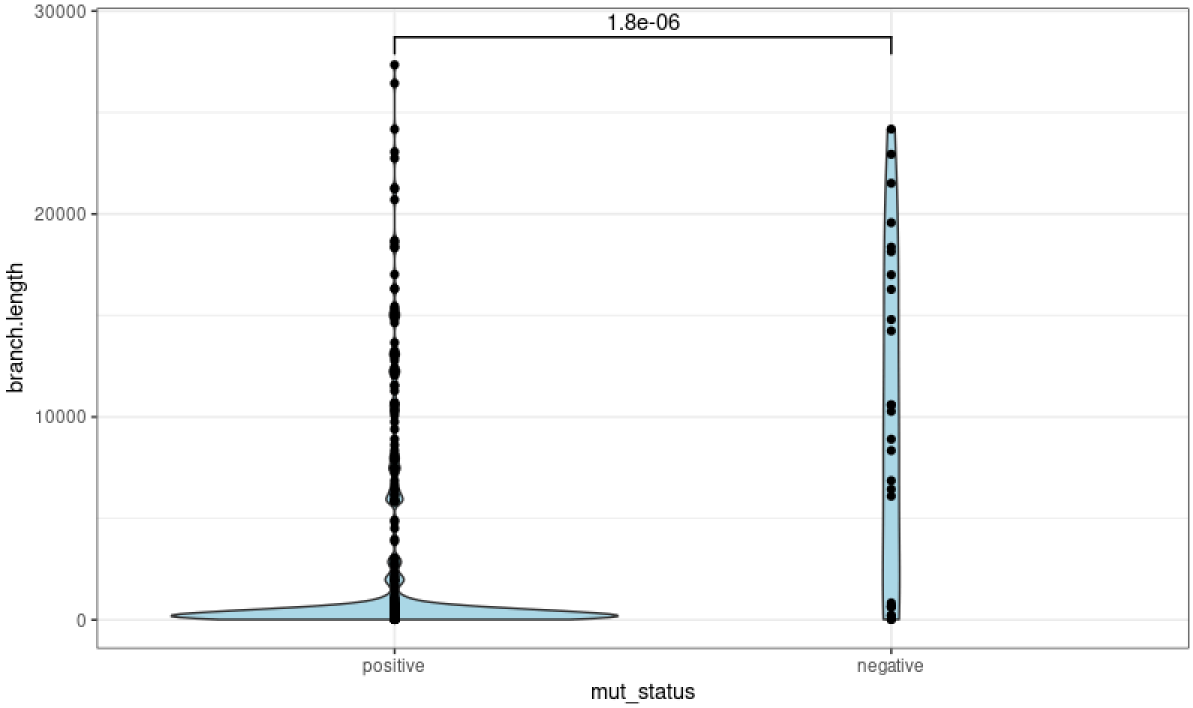

Figure S19. Strains without detectable mutacins have significantly longer branch lengths. BEAST estimated branch length for each analysed genome is plotted against mutacin status (whether mutacins are detected or not). The difference between these branch lengths was assessed using a wilcoxon test, as implemented in the R package ggsignif; the p value from this test is printed above the plotted comparison.

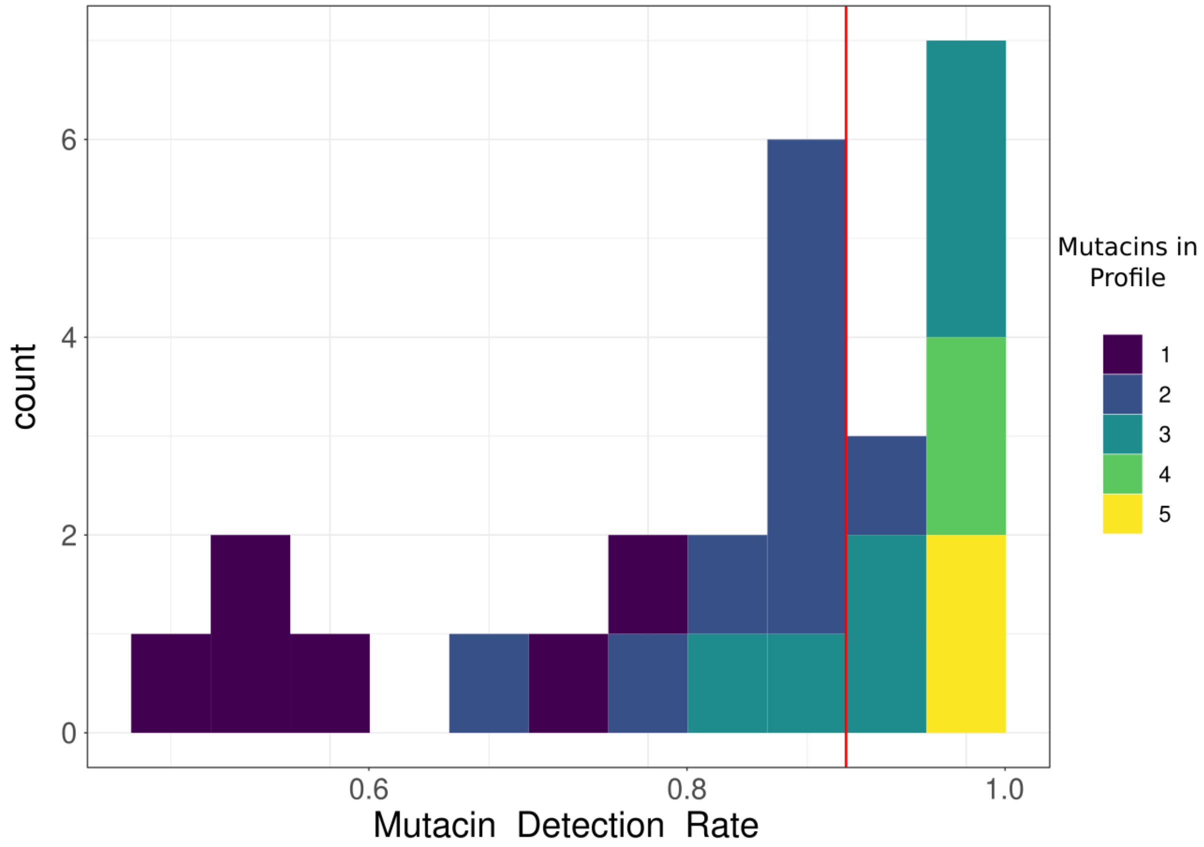

Figure S20. Mutacin Detection Rate from simulations.

Histogram of mutacin profile detection rate in 5% bins, coloured by the number of mutacins in those profiles. Red line marks 90% detection rate. Detection is from simulated 0.4X data - see **Table S7** for underlying counts.

References

Ajdić D, McShan WM, McLaughlin RE, Savić G, Chang J, Carson MB, Primeaux C, Tian R, Kenton S, Jia H, et al. 2002. Genome sequence of Streptococcus mutans UA159, a cariogenic dental pathogen. *Proc. Natl. Acad. Sci. U. S. A.* 99:14434–14439.

Alneberg J, Bjarnason BS, de Bruijn I, Schirmer M, Quick J, Ijaz UZ, Lahti L, Loman NJ, Andersson AF, Quince C. 2014. Binning metagenomic contigs by coverage and composition. *Nat. Methods* 11:1144–1146.

Bouckaert RR, Drummond AJ. 2017. bModelTest: Bayesian phylogenetic site model averaging and model comparison. *BMC Evol. Biol.* 17:42.

Bouckaert R, Vaughan TG, Barido-Sottani J, Duchêne S, Fourment M, Gavryushkina A, Heled J, Jones G, Kühnert D, De Maio N, et al. 2019. BEAST 2.5: An advanced software platform for Bayesian evolutionary analysis. *PLoS Comput. Biol.* 15:e1006650.

Bravo-Lopez M, Villa-Islas V, Rocha Arriaga C, Villaseñor-Altamirano AB, Guzmán-Solís A, Sandoval-Velasco M, Wesp JK, Alcantara K, López-Corral A, Gómez-Valdés J, et al. 2020. Paleogenomic insights into the red complex bacteria Tannerella forsythia in Pre-Hispanic and Colonial individuals from Mexico. *Philos. Trans. R. Soc. Lond. B Biol. Sci.* 375:20190580.

Buchfink B, Reuter K, Drost H-G. 2021. Sensitive protein alignments at tree-of-life scale using DIAMOND. *Nat. Methods* 18:366–368.

Cantu VA, Sadural J, Edwards R. 2019. PRINSEQ++, a multi-threaded tool for fast and efficient quality control and preprocessing of sequencing datasets. *PeerJ Preprints* 7:e27553v1.

Cassidy LM, Maoldúin RÓ, Kador T, Lynch A, Jones C, Woodman PC, Murphy E, Ramsey G, Dowd M, Noonan A, et al. 2020. A dynastic elite in monumental Neolithic society. *Nature* 582:384–388.

Chaumeil P-A, Mussig AJ, Hugenholtz P, Parks DH. 2019. GTDB-Tk: a toolkit to classify genomes with the Genome Taxonomy Database. *Bioinformatics* 36:1925–1927.

Croucher NJ, Page AJ, Connor TR, Delaney AJ, Keane JA, Bentley SD, Parkhill J, Harris SR. 2015. Rapid phylogenetic analysis of large samples of recombinant bacterial whole genome sequences using Gubbins. *Nucleic Acids Res.* 43:e15.

Dabdoub SM. 2016. kraken-biom: Enabling interoperative format conversion for Kraken results. Available from: https://github.com/smdabdoub/kraken-biom

Drummond AJ, Ho SYW, Phillips MJ, Rambaut A. 2006. Relaxed phylogenetics and dating with confidence. *PLoS Biol.* 4:e88.

Drummond AJ, Rambaut A, Shapiro B, Pybus OG. 2005. Bayesian coalescent inference of past population dynamics from molecular sequences. *Mol. Biol. Evol.* 22:1185–1192.

Fellows Yates JA, Velsko IM, Aron F, Posth C, Hofman CA, Austin RM, Parker CE, Mann AE, Nägele K, Arthur KW, et al. 2021. The evolution and changing ecology of the African hominid oral microbiome. *Proc. Natl. Acad. Sci. U. S. A.* 118:e2021655118.

Guellil M. 2021. MeriamGuellil/aDNA-BAMPlotter: aDNA-BAMPlotter. Available from: https://zenodo.org/record/5676093

Harris SR. 2018. SKA: Split Kmer Analysis Toolkit for Bacterial Genomic Epidemiology. *bioRxiv* [Internet]:453142. Available from: https://www.biorxiv.org/content/10.1101/453142v1

Hijmans RJ. 2022. Spherical Trigonometry [R package geosphere version 1.5-18]. Available from: https://CRAN.R-project.org/package=geosphere

Hoang DT, Chernomor O, von Haeseler A, Minh BQ, Vinh LS. 2018. UFBoot2: Improving the Ultrafast Bootstrap Approximation. *Mol. Biol. Evol.* 35:518–522.

Honap TP, Monroe CR, Johnson SJ, Jacobson DK, Abin CA, Austin RM, Sandberg P, Levine M, Sankaranarayanan K, Lewis CM Jr. 2023. Oral metagenomes from Native American Ancestors reveal distinct microbial lineages in the pre-contact era. *Am J Biol Anthropol* 1-15. Available from: http://dx.doi.org/10.1002/ajpa.24735

Hübler R, Key FM, Warinner C, Bos KI, Krause J, Herbig A. 2019. HOPS: automated detection and authentication of pathogen DNA in archaeological remains. *Genome Biol.* 20:280.

Human Microbiome Project Consortium. 2012. Structure, function and diversity of the healthy human microbiome. *Nature* 486:207–214.

Johnston ER, Rodriguez-R LM, Luo C, Yuan MM, Wu L, He Z, Schuur EAG, Luo Y, Tiedje JM, Zhou J, et al. 2016. Metagenomics Reveals Pervasive Bacterial Populations and Reduced Community Diversity across the Alaska Tundra Ecosystem. *Front. Microbiol.* 7:579.

Kalyaanamoorthy S, Minh BQ, Wong TKF, von Haeseler A, Jermiin LS. 2017. ModelFinder: fast model selection for accurate phylogenetic estimates. *Nat. Methods* 14:587–589.

Knights D, Kuczynski J, Charlson ES, Zaneveld J, Mozer MC, Collman RG, Bushman FD, Knight R, Kelley ST. 2011. Bayesian community-wide culture-independent microbial source tracking. *Nat. Methods* 8:761–765.

Ksiazek M, Mizgalska D, Eick S, Thøgersen IB, Enghild JJ, Potempa J. 2015. KLIKK proteases of Tannerella forsythia: putative virulence factors with a unique domain structure. *Front. Microbiol.* 6:312.

Li D, Liu C-M, Luo R, Sadakane K, Lam T-W. 2015. MEGAHIT: an ultra-fast single-node solution for large and complex metagenomics assembly via succinct de Bruijn graph. *Bioinformatics* 31:1674–1676.

Li H, Durbin R. 2009. Fast and accurate short read alignment with Burrows-Wheeler transform. *Bioinformatics* 25:1754–1760.

Li H, Handsaker B, Wysoker A, Fennell T, Ruan J, Homer N, Marth G, Abecasis G, Durbin R,

1000 Genome Project Data Processing Subgroup. 2009. The Sequence Alignment/Map format and SAMtools. *Bioinformatics* 25:2078–2079.

Lloyd-Price J, Mahurkar A, Rahnavard G, Crabtree J, Orvis J, Hall AB, Brady A, Creasy HH, McCracken C, Giglio MG, et al. 2017. Strains, functions and dynamics in the expanded Human Microbiome Project. *Nature* 550:61–66.

Lu J, Breitwieser FP, Thielen P, Salzberg SL. 2017. Bracken: Estimating species abundance in metagenomics data. *PeerJ Computer Science* 2017:e104.

Martin M. 2011. Cutadapt removes adapter sequences from high-throughput sequencing reads. *EMBnet.journal* 17:10.

McKenna A, Hanna M, Banks E, Sivachenko A, Cibulskis K, Kernytsky A, Garimella K, Altshuler D, Gabriel S, Daly M, et al. 2010. The Genome Analysis Toolkit: A MapReduce framework for analyzing next-generation DNA sequencing data. *Genome Res.* 20:1297–1303.

Meyer M, Kircher M. 2010. Illumina sequencing library preparation for highly multiplexed target capture and sequencing. *Cold Spring Harb. Protoc.* 5:db.prot5448.

Minh BQ, Schmidt HA, Chernomor O, Schrempf D, Woodhams MD, von Haeseler A, Lanfear R. 2020. IQ-TREE 2: New Models and Efficient Methods for Phylogenetic Inference in the Genomic Era. *Mol. Biol. Evol.* 37:1530–1534.

Okonechnikov K, Conesa A, García-Alcalde F. 2015. Qualimap 2: advanced multi-sample quality control for high-throughput sequencing data. *Bioinformatics* 32:btv566.

Page AJ, Cummins CA, Hunt M, Wong VK, Reuter S, Holden MTG, Fookes M, Falush D, Keane JA, Parkhill J. 2015. Roary: rapid large-scale prokaryote pan genome analysis. *Bioinformatics* 31:3691–3693.

Page AJ, Taylor B, Delaney AJ, Soares J, Seemann T, Keane JA, Harris SR. 2016. SNP-sites: rapid efficient extraction of SNPs from multi-FASTA alignments. *Microb Genom* 2:e000056.

Paradis E, Schliep K. 2019. ape 5.0: an environment for modern phylogenetics and evolutionary analyses in R. *Bioinformatics* 35:526–528.

Parks DH, Imelfort M, Skennerton CT, Hugenholtz P, Tyson GW. 2015. CheckM: assessing the quality of microbial genomes recovered from isolates, single cells, and metagenomes. *Genome Res.* 25:1043–1055.

Philips A, Stolarek I, Handschuh L, Nowis K, Juras A, Trzciński D, Nowaczewska W, Wrzesińska A, Potempa J, Figlerowicz M. 2020. Analysis of oral microbiome from fossil human remains revealed the significant differences in virulence factors of modern and ancient Tannerella forsythia. *BMC Genomics* 21:402.

Purcell S, Neale B, Todd-Brown K, Thomas L, Ferreira MAR, Bender D, Maller J, Sklar P, de

Bakker PIW, Daly MJ, et al. 2007. PLINK: A Tool Set for Whole-Genome Association and Population-Based Linkage Analyses. *Am. J. Hum. Genet.* 81:559–575.

Rambaut A, Lam TT, Max Carvalho L, Pybus OG. 2016. Exploring the temporal structure of heterochronous sequences using TempEst (formerly Path-O-Gen). *Virus Evol* 2:vew007.

R Core Team. 2023. R: A Language and Environment for Statistical Computing. Available from: https://www.R-project.org/

Renaud G, Hanghøj K, Willerslev E, Orlando L. 2017. gargammel: a sequence simulator for ancient DNA. *Bioinformatics* 33:577–579.

Salter SJ, Cox MJ, Turek EM, Calus ST, Cookson WO, Moffatt MF, Turner P, Parkhill J, Loman NJ, Walker AW. 2014. Reagent and laboratory contamination can critically impact sequence-based microbiome analyses. *BMC Biol.* 12:87.

Schubert M, Lindgreen S, Orlando L. 2016. AdapterRemoval v2: rapid adapter trimming, identification, and read merging. *BMC Res. Notes* 9:88.

Seemann T. 2014. Prokka: rapid prokaryotic genome annotation. *Bioinformatics* 30:2068–2069.

Seguin-Orlando A, Donat R, Der Sarkissian C, Guilaine J, Orlando L. 2021. Heterogeneous Hunter-Gatherer and Steppe- Related Ancestries in Late Neolithic and Bell Beaker Genomes from Present-Day France. *Curr. Biol.*:1–12.

Smith MR, Wickham H. 2023. ms609/TreeTools: v1.9.2. Zenodo Available from: https://zenodo.org/record/3522725

Socransky SS, Haffajee AD, Cugini MA, Smith C, Kent RL Jr. 1998. Microbial complexes in subgingival plaque. *J. Clin. Periodontol.* 25:134–144.

Watanabe A, Kawada-Matsuo M, Le MN-T, Hisatsune J, Oogai Y, Nakano Y, Nakata M, Miyawaki S, Sugai M, Komatsuzawa H. 2021. Comprehensive analysis of bacteriocins in Streptococcus mutans. *Sci. Rep.* 11:12963.

Willmann C, Mata X, Hanghoej K, Tonasso L, Tisseyre L, Jeziorski C, Cabot E, Chevet P, Crubézy E, Orlando L, et al. 2018. Oral health status in historic population: Macroscopic and metagenomic evidence.Caramelli D, editor. *PLoS One* 13:e0196482.

Wood DE, Lu J, Langmead B. 2019. Improved metagenomic analysis with Kraken 2. *Genome Biol.* 20:257.

Woodman P, Dowd M, Fibiger L, Carden RF. 2017. Archaeological excavations at Killuragh Cave, Co. Limerick: a persistent place in the landscape from the Early Mesolithic to the Late Bronze Age. *The Journal of Irish Archaeology* 26:1–32.

Zeng H, Chan Y, Gao W, Leung WK, Watt RM. 2021. Diversity of Treponema denticola and Other Oral Treponeme Lineages in Subjects with Periodontitis and Gingivitis. *Microbiol Spectr* 9:e0070121.
